# Serine protease PRSS55 is crucial for male mouse fertility via affecting sperm migration and sperm–egg binding

**DOI:** 10.1007/s00018-018-2878-9

**Published:** 2018-07-21

**Authors:** Xuan Shang, Chunling Shen, Jianbing Liu, Lingyun Tang, Hongxin Zhang, Yicheng Wang, Wenting Wu, Jun Chi, Hua Zhuang, Jian Fei, Zhugang Wang

**Affiliations:** 1 State Key Laboratory of Medical Genomics, Research Center for Experimental Medicine, Shanghai Ruijin Hospital Affiliated to Shanghai Jiao Tong University School of Medicine, Shanghai, 200025 China; 2 Shanghai Research Center for Model Organisms, Shanghai, 201203 China; 3 Changzhou Woman and Children Health Hospital Affiliated with Nanjing Medical University, Changzhou, 213003 China

**Keywords:** Serine protease PRSS55, Knockout mouse model, Male infertility, Sperm migration, Sperm–egg recognition/binding

## Abstract

Testis-specific PRSS55 is a highly conserved chymotrypsin-like serine protease among mammalian species. So far, the physiological function of PRSS55 remains unknown. Here, we show that PRSS55 is a GPI-anchored membrane protein, specifically expressed in adult mouse testis and mainly observed in the luminal side of seminiferous tubules and sperm acrosome. Mice deficient for *Prss55* develop male infertile with normal reproduction-related parameters observed. Interestingly, in vivo fertilization rate of *Prss55*^−*/*−^ males is dramatically decreased, possibly due to incapable migration of *Prss55*^−*/*−^ sperm from uterus into oviduct. However, in vitro fertilization rate has no difference between two genotypes although *Prss55*^−*/*−^ sperm presents defective recognition/binding to zona-intact or zona-free oocytes. Further study reveals that mature ADAM3 is almost undetectable in *Prss55*^−*/*−^ sperm, while precursor ADAM3 remains unchanged in the testis. However, it is shown that ADAM3 has no interaction with PRSS55 by immunoprecipitation with anti-PRSS55 antibody. The expression levels of several proteins known to be related to the observed phenotypes remain comparable between wt and *Prss55*^−*/*−^ mice. Moreover, we found that *Prss55* deficiency has no effect on PRSS37 or vice versa albeit two mutant males share almost the same phenotypes. Microarray analysis reveals a total of 72 differentially expressed genes in *Prss55*^−*/*−^ testis, most of which are associated with cellular membrane and organelle organization, protein transport and complex assembly, and response to stimulus and signaling. In conclusion, we have demonstrated that PRSS55 plays vital roles in regulating male fertility of mice, including in vivo sperm migration and in vitro sperm–egg interaction, possibly by affecting the maturation of ADAM3 in sperm and the expression of multiple genes in testis.

**Electronic supplementary material:**

The online version of this article (10.1007/s00018-018-2878-9) contains supplementary material, which is available to authorized users.

## Introduction

The in vivo fertilization of mammalians is a complex process containing several coordinated events. After sperm are deposited into the female reproductive tract at coitus, they must undergo capacitation, migration and penetration through the cumulus cells as well as the zona pellucida (ZP) of the oocytes to achieve successful fertilization. Although many millions of sperm are deposited into the female reproductive tract, only a few hundred or less will actually reach the eggs. The passage of sperm through the female reproductive tract is finely regulated to select the sperm with normal morphology, vigorous motility and sound fertilizing capability [1, 2]. However, the molecular mechanisms that regulate the in vivo migration of sperm in the female genital tract are not yet fully clarified.

Excitingly, over the past two decades, a number of genes have been verified to play essential roles in regulating this process using genetically manipulated mouse models, including *Clgn* [3, 4], *Calr3* [5], *Adam1a* [6], *Adam2* [7], *Adam3* [8, 9], t-*Ace* [10], *Tpst2* [11, 12], *Pdilt* [13], *Rnase10* [14], *Pmis2* [15], *Prss37* [16], *Tex101* [17, 18] and *Ly6k* [18]. These gene-encoded proteins are varied in cellular component and molecular function. For example, *Clgn* and *Calr3* encode testis-specific endoplasmic reticulum (ER) chaperones; ADAM1A, ADAM2 and ADAM3 are three members of the ADAM family that is composed of structurally related cell surface proteins proposed to have cell adhesion activity; TPST2 catalyzes post-translational protein tyrosine O-sulfation; PDILT cooperates with CALR3 in the ER and functions in the disulfide-bond formation; *Rnase10*, a pancreatic *Rnase A* homolog, encodes a secreted proximal epididymal protein in the mouse; PRSS37 is a putative trypsin-like serine protease exclusively expressed during a short time range of late spermiogenesis and does not exist in mature mouse sperm; TEX101/LY6K, a glycosylphosphatidylinositol (GPI)-anchored protein complex specifically expressed in testicular germ cells, interacts with precursor ADAM3 in the testis and is a tACE-specific substrate. It is of note that ADAM3 is absent or abnormally located in the mature sperm of all the above mentioned mouse lines. Its deficiency in mice results in male infertility, defective sperm-ZP binding and impaired sperm migration into oviduct. However, the molecular mechanisms regarding the maturation of ADAM3 in the sperm, especially the functional network among the above-mentioned genes are not fully understood and whether other genes are involved in this process remains to be investigated. The ongoing efforts to identify such genes are of great significance since they may be potential causes of unexplained male infertility (UMI) in men and thus be potential targets for the development of new drugs for both the treatment of UMI and male contraception.

Human PRSS55, also known as T-SP1, is first identified as a novel member in the group of membrane-anchored chymotrypsin (S1)-like serine proteases predominantly expressed in testis [19]. It is located on chromosome 8p23.1 and composed of 10 exons spanning 28.6 kb. Different variants of human *PRSS55* mRNA are produced due to alternative splicing. It is reported that PRSS55 is also expressed in prostate cancer and some ovarian cancer tissues, indicating its important roles in spermatogenesis and tumorigenesis [19, 20]. Mouse *Prss55*, located on chromosome 14D1 and composed of 7 exons, encodes a 321-aa protein. Protein sequence analysis reveals that PRSS55 is highly conserved among various species (Supplementary Fig. S1), strongly suggesting an important evolutionarily conserved roles, especially in male mammalian reproduction. However, the physiological function of PRSS55 remains unknown so far. In the present paper, we have characterized the biochemical and physiological properties of PRSS55 in vitro and in vivo, demonstrating that PRSS55 is a GPI-anchored membrane protein, predominantly expressed in adult mouse testis. It is mainly distributed in the luminal margin of seminiferous tubules as well as sperm acrosome region. Functionally, it is required for male fertility in mice and is involved in the processes of sperm migration into oviduct and sperm-zona recognition/binding. These findings disclose an important function of PRSS55, further implicating that PRSS55 may play a pathogenic role in the development of UMI and act as a potential drug target for UMI patients or male contraception.

## Materials and methods

### Mice and genotyping

*Prss55* knockout (KO) mice were generated by homologous recombination and maintained on a mixed 129 Sv/C57BL/6 background. Heterozygous mice were intercrossed to generate the offspring with three different genotypes. Triple primer PCR strategy was designed for routine genotyping using the following amplification conditions: 95 °C for 4 min and 35 cycles of 94 °C for 30 s, 60 °C for 30 s, 72 °C for 40 s, and a 10-min incubation at 72ºC at the end of the run. PCR products were resolved on 1.5% agarose gels and the products derived from wild type (wt) and targeted alleles are 861 and 486-bp, respectively. All mice were housed under specific pathogen free conditions at a constant room temperature of 22–24 °C with a 12-h light/dark cycle, with free access to a diet of regular chow and water. All research protocols involving animal experiments were approved by the Institutional Animal Care and Use Committee of Shanghai Research Center for Model Organisms (Permit Number: 2014-0020).

### Analyses of mRNA expression

Total RNAs extracted from mouse tissues using Trizol Reagent (Roche) were reverse transcribed using Advantage RT-for-PCR Kit (Takara, Dalian, China) according to the manufacturers’ instructions. cDNAs were amplified using specific sets of primers listed in Supplementary Table S1 for semi- or real-time RT-PCR. Semi-RT-PCR products were separated by electrophoresis on 1.5% agarose gels and visualized by ethidium bromide staining. Real-time PCR was performed by Mastercycler ep realplex (Eppendorf) using SYBR Premix Ex Taq Kit (Takara). Resolution of the product of interest from nonspecific product amplification was achieved by melt curve analysis. Gene expression levels are normalized to *Actb* content using the 2^−ΔΔCt^ or 2^−ΔCt^ method [21].

### Preparation of mouse polyclonal anti-PRSS55 antiserum

An 918-bp DNA fragment encoding the mature peptide (17-321 aa) of mouse PRSS55 was synthesized after codon optimization and was inserted into the *Nde*I-*Xho*I sites of pET-22b (+) vector (Novagen, Madison, WI) for expression in *Escherichia coli* BL21 (DE3). Recombinant mouse PRSS55 (0.1 mg in 50 μl) was emulsified by homogenization with an equal volume of Freund’s complete adjuvant (Sigma) and injected into wt or *Prss55*^−*/*−^ male mice intraperitoneally. Three booster immunizations were performed on days 14, 35 and 56 by injection of 0.1 mg recombinant mouse PRSS55 emulsified with Freund’s incomplete adjuvant. Mice were killed on day 66 and whole blood was collected for the preparation of anti-PRSS55 antiserum.

### Preparation of protein extracts

Proteins from mouse tissues (including testis, caput, corpus and cauda epididymis) were extracted in PBS containing 1% SDS (PBS/SDS) supplemented with protease and phosphatase inhibitor cocktails. After a short sonication, samples were incubated at room temperature for 1 h and then centrifuged at 16,000×g for 10 min. The resulting supernatants were applied for immunoblot analysis. Proteins of mature sperm collected from the cauda epididymides of mice were extracted by 1 mM HCl containing 1% SDS (HCl/SDS) supplemented with protease and phosphatase inhibitor cocktails at room temperature for 1 h. After centrifugation at 16,000×g for 10 min, the supernatants were harvested as sperm protein extracts. Proteins from different fractions of testis were obtained by membrane, nuclear and cytoplasmic protein extraction kit (Shenggong, Shanghai, China) according to the manufacturer’s instruction. Protein concentration was determined with a BCA protein assay kit (Pierce, Rockford, IL, USA).

### Western blotting

Protein samples were supplemented 5 × concentrated SDS-PAGE sample buffer containing β-mercaptoethanol and boiled at 100 °C for 5 min. Then, they were separated on 10% SDS-PAGE gels and transferred onto nitrocellulose membranes (GE). Membranes were blocked with 5% nonfat milk for 1 h, followed by incubation with the primary antibodies: anti-PRSS55, anti-GAPDH (2118S, Cell signaling technology), anti-Lamin A/C (2032T, Cell signaling technology), anti-NaKATPase (3010S, Cell signaling technology), anti-uPAR (ab103791, Abcam), anti-ACTB (sc-47778, Santa Cruz), anti-ADAM3 (sc-365288, Santa Cruz), anti-ADAM2 (MAB19292, Millipore), anti-CLGN (ab171971, Abcam), anti-PDILT (ab116182, Abcam), anti-PRSS37 (HPA020541, Sigma-Aldrich), anti-TEX101 (ab69522, Abcam), anti-tACE (AF1513, R&D Systems), anti-ATP5A1 (14676-1-AP, Proteintech) and anti-GFP (G1544, Sigma). To visualize specific protein bands, secondary antibodies conjugated with IRdye800CW (LI-COR, Lincoln, USA) were used and membranes were scanned by Odyssey Infrared Imager (LI-COR). ACTB, GAPDH or ATP5A1 were used as protein loading controls.

### Immunoprecipitation (IP)

IP was performed according to the previously published protocol [13] with several modifications. Magnetic bead separation (Protein G-Magnetic Beads; MBL) was used. Testicular cells were solubilized with 1 ml of ice-cold lysis buffer [PBS, 1% SDS, and 1% protease inhibitor mixture (Roche)] and then centrifuged at 16,000×g for 10 min at 4 °C. 1 mg of testis lysate was diluted with PBS to reduce the concentration of SDS to 0.2%, incubated with 100 μl of protein G-conjugated beads for 1 h at 4 °C to pre-clear the lysate and then incubated with 2 μg of each antibody or negative control IgG and 100 μl of protein G beads at 4 °C overnight. After rinsing five times with 500 μl of the wash buffer (PBS, 0.1% SDS), 60 μl 1 × SDS sample buffer was added to the tubes and heated to 99 °C, and eluate fractions were collected for analysis by SDS-PAGE.

### Cell culture and transfections

HEK293 cells were cultured in standard commercial DMEM medium (Gibco) containing 10% (vol/vol) FBS at 37 °C with 5% CO_2_. Full-length *Prss55* cDNA was amplified from wt testis cDNA using primers pEGFP-Prss55-F and pEGFP-Prss55-R (Supplementary Table S1). A Kozak sequence GCCACC was added before the translation initiation site. The sequence was sub-cloned into the *Xho*I-*Hind*III sites of the eukaryotic expression vector pEGFP-N2 (Invitrogen) and confirmed by sequencing. Purified pEGFP-N2 null vector and pEGFP-Prss55 vector were transfected into HEK293 cells with Lipofectamine 3000 transfection reagent (Invitrogen). 48 h after transfection, the localization of EGFP and EGFP fusion protein was visualized under a confocal microscope (Fluo View FV10i, Olympus, Tokyo, Japan). For Western blot analysis, cells were collected 48 h after transfection and assayed.

### Phosphatidylinositol-specific phospholipase C (PI-PLC) digestion of testicular cells, cultured cells and sperm suspension

PI-PLC digestion of cells was performed according to the previously published protocols [22, 23] with minor modifications. Briefly, equal aliquots of testicular cells and sperm suspension in PBS were treated with various concentrations (0, 1, 2.5, 5 U/ml) of PI-PLC from *Bacillus cereus* (Sigma) for 1 h at 37 °C with mixing every 15 min. The HEK293 cells transfected with pEGFP or pEGFP-Prss55 for 48 h were treated with/without 2.5 U/ml PI-PLC for 1 h at 37 °C. The protein levels of PRSS55 in supernatants and/or in pellets were immunodetected by Western blotting. Urokinase-type plasminogen activator receptor (uPAR), a representative GPI-anchored protein in germ cells, was used as a positive control [24, 25].

### Gradual release of proteins during acrosome reaction (AR) induced by A23187

Cauda epididymal mouse sperm were capacitated in TYH medium with 0.4% BSA at 37 °C for 1 h. The sperm suspension (1 ~ 2 × 10^7^ sperm) was divided into four aliquots and incubated with A23187 (final concentration of 10 μM) for 0, 10, 30, and 60 min, respectively. The treated sperm were centrifuged at 10,000×g for 5 min to obtain supernatants, which contained proteins released by AR, and sperm pellets, which contained remaining proteins on sperm. The sperm pellets were washed with PBS for three times and then lysed in HCl/SDS supplemented with protease and phosphatase inhibitor cocktails at room temperature for 1 h.

### Proteinase *K* digestion of sperm suspension and testicular cells

Proteinase *K* digestion of sperm suspension and testicular cells was performed as previously described [26] with minor modifications. Testicular cells and sperm from pAcr-SP-NTP-EGFP transgenic mice [27] were suspended in PBS (2 × 10^7^ cells/ml), and 90 μl aliquots from each cell suspension were incubated with 10 μl of various concentrations of proteinase *K* (0, 10, 20, 50, 100 μg/ml) for 30 min at 37 °C. The proteinase *K* digestion was terminated by adding 2 μl of 100 mM phenylmethanesulfonyl fluoride (PMSF) followed by incubation at room temperature for 15 min. The digested cell samples were centrifuged at 10,000×g for 5 min at 4 °C to obtain supernatants. The pellets were washed with PBS for three times and then lysed in 100 μl PBS/SDS or HCl/SDS containing 2 μl of 100 mM PMSF for 30 min on ice. The resulting supernatants obtained by centrifugation at 20,000×g for 10 min at 4 °C were regarded as pellet extracts.

### Histological and immunofluorescence (IF) analyses

Testes from adult age-matched mice with different genotypes were isolated and weighted. Paraffin-embedded tissue sections of testis and epididymis were prepared as described [16]. Sperm smears were prepared by spotting sperm isolated from cauda epididymis onto glass slides, air dried and fixed in 4% paraformaldehyde. For histological analysis, samples were stained with hematoxylin and eosin (H&E). Transmission electronic microscopy (TEM) was performed as previously described [16]. For IF, samples were washed in 1 × PBS, blocked in 5% normal goat serum, and incubated with anti-PRSS55 antiserum (1:100 dilution) overnight at 4 °C. After three washes in PBS for 5 min each, Alexa Fluo 594-conjugated goat anti-mouse secondary antibody was added and allowed incubation for 1 h at room temperature. Nuclei were visualized by DAPI (Invitrogen) conterstain. Alexa Fluo 488-conjugated lectin peanut agglutinin (PNA, Molecular Probes) was used to label sperm acrosome. Slides were mounted in fluorescence mounting medium (DAKO, Glostrup, Denmark), coverslipped and examined under a fluorescence microscope (Nikon Eclipse 90i).

### Male fertility evaluation

Adult male mice of three genotypes (*n* ≥ 5 males each group) were caged with wt females at a 1:2 male to female sex ratio for a month. Plugs were checked every day and plugged females were removed and replaced with new ones. The number of total females, plugged females, litters and offsprings was counted to calculate the frequency of copulatory plug (FCP) and frequency of conception (FC). The in vivo fertilization rate was also used to evaluate the fertility of males. wt and *Prss55*-KO males (*n* ≥ 5 males each group) were mated with wt females superovulated by intraperitoneal injection of 5 units of pregnant mare serum gonadotrophin (PMSG) followed by 5 units of human chorionic gonadotropin (hCG) 46–48 h later. The oviducts of plugged females were excised 45 h after hCG injection and the oocytes as well as the 2-cell embryos were recovered. The percentage of the 2-cell embryos was regarded as in vivo fertilization rate.

### Sperm migration in the female reproductive tract

Sperm migration in the female reproductive tract was assayed by counting sperm present in the oviduct 3.5 h after coitus and by tracing the migration of EGFP-expressing sperm from uterus into oviduct under the stereomicroscope SMZ1500 (Nikon) under excitation light. EGFP-expressing *Prss55*-KO males (*Prss55*^−*/*−^*EGFP*^*tg/*+^) were generated by crossing *Prss55*^−*/*−^ females with pAcr-SP-NTP-EGFP transgenic mice [27] followed by mating between siblings.

### Computer-assisted semen analysis (CASA)

Parameters of sperm motility were quantified by CASA using an integrated visual optical system software (Hamilton-Thorne Biosciences). The procedures of the detection method have been previously described [16].

### Acrosome reaction and activity of acrosomal enzymes

Sperm from the cauda epididymides were capacitated in TYH medium with 0.4% BSA at 37 °C for 1 h. Calcium ionophore A23187 (Sigma) at a final concentration of 10 μM was used to induce AR. The occurrence of AR was examined by PNA staining of sperm smears. The activity of acrosomal enzymes released by AR was assayed using different fluorescent substrates: 4-methylumbelliferyl phosphate (MUP), t-butyloxycarbonyl (Boc)-Ala-Gly-Pro-Arg-4-methylcoumaryl-7-amide (MCA) (AGPR), Boc-Val-Pro-Arg-MCA (VPR), and Boc-Leu-Thr-Arg-MCA (LTR). The reaction mixture with MUP substrate was consisted of 25 mM citric acid (pH 4.5), 0.03 mg/ml MUP, and an appropriate amount of acrosomal enzymes, and the reaction was terminated by addition of equal volume of 0.4 M glycine (pH 10.4). The reaction mixture with the other substrates was consisted of 50 mM Tris·HCl (pH 8.0), 10 μM substrate, and an appropriate amount of acrosomal enzymes, and the reaction was terminated by the addition of three volumes of 0.1 M acetate buffer (pH 4.3). The fluorophores released after enzymatic reaction were detected with excitation at 380 nm and emission at 460 nm.

### Sperm–egg binding assay and assessment of PRSS55 on sperm–egg binding ability

ZP-intact oocytes were obtained by treating the oocyte-cumulus complexes (OCCs) isolated from superovulated females with 0.01% (w/v) hyaluronidase (Sigma). ZP-free oocytes were obtained by further treating the ZP-intact oocytes with acidic Tyrode solution (Sigma). ZP-intact and ZP-free oocytes were incubated with capacitated cauda epididymal sperm for 30 min and sperm–egg binding was observed using an IX71 microscope (Olympus, Tokyo, Japan). The antibody blocking assay was performed as previously described [28, 29] with minor modifications. Briefly, different volumes of PRSS55 antibody (1, 2, and 3 μl) or pre-immune serum (0, 1, 2, and 3 μl) were pre-incubated with zona-intact or zona-free oocytes for 30 min in M16 medium and then the capacitated sperm were added to co-incubate for another 30 min in 200 μl drops of M16 medium and sperm–egg binding was observed using an IX71 microscope (Olympus, Tokyo, Japan).

### In vitro fertilization (IVF) assay

Cauda epididymal sperm were freshly isolated and incubated in human tube fluid (HTF) medium [30] with 0.4% BSA at 37 °C under 5% CO_2_ in air for 1 h to allow capacitation. OCCs were isolated from superovulated female mice 14 h after hCG injection and placed in a 200 μl drop of HTF medium. An aliquot of capacitated sperm from wt and *Prss55*^−*/*−^ males was mixed with OCCs and incubated at 37 °C under 5% CO_2_. After 6 h, oocytes were washed and transferred to potassium simplex medium (KSOM) [31] for culture to later stages. The percentage of the 2-cell embryos examined 24–30 h later was regarded as IVF rate.

### Microarray analysis

Total RNAs were extracted from testes of wt and *Prss55*^−*/*−^ mice (*n* = 3 each genotype, 6–8 weeks old), purified using RNeasy mini kit (Cat#74106, Qiagen, GmBH, Germany) following the manufacturer’s instructions and checked for an RIN number to inspect RNA integration by an Agilent Bioanalyzer 2100 (Agilent technologies, Santa Clara, CA, USA). The sample labelling, hybridization, staining and scanning procedures were carried out at the National Engineering Research Center of Shanghai using the whole mouse genome oligo microarray (4 × 44 k, Agilent). The experimental data were normalized by quantile algorithm, Gene-Spring software GX 12.6.1 (Agilent). The differentially expressed (DE) transcripts were determined based on Student’s *t* test (*P* < 0.05) and fold change. All DE genes were clustered by Hierarchical Clustering method. Original data are available in the Gene Expression Omnibus (GEO) at the National Center for Biotechnology Information (NCBI) website (Accession Number GSE107350).

### Statistical analysis

All data are expressed in terms of mean values ± standard error (SE) (*n* ≥ 3), unless otherwise stated. The differences between two groups were analyzed using two-tailed Student’s t test. For all the statistical tests, *p* values less than 0.05 were considered statistically significant.

## Results

### PRSS55 is a GPI-anchored membrane protein specifically expressed in adult mouse testis

**Fig. 1:**
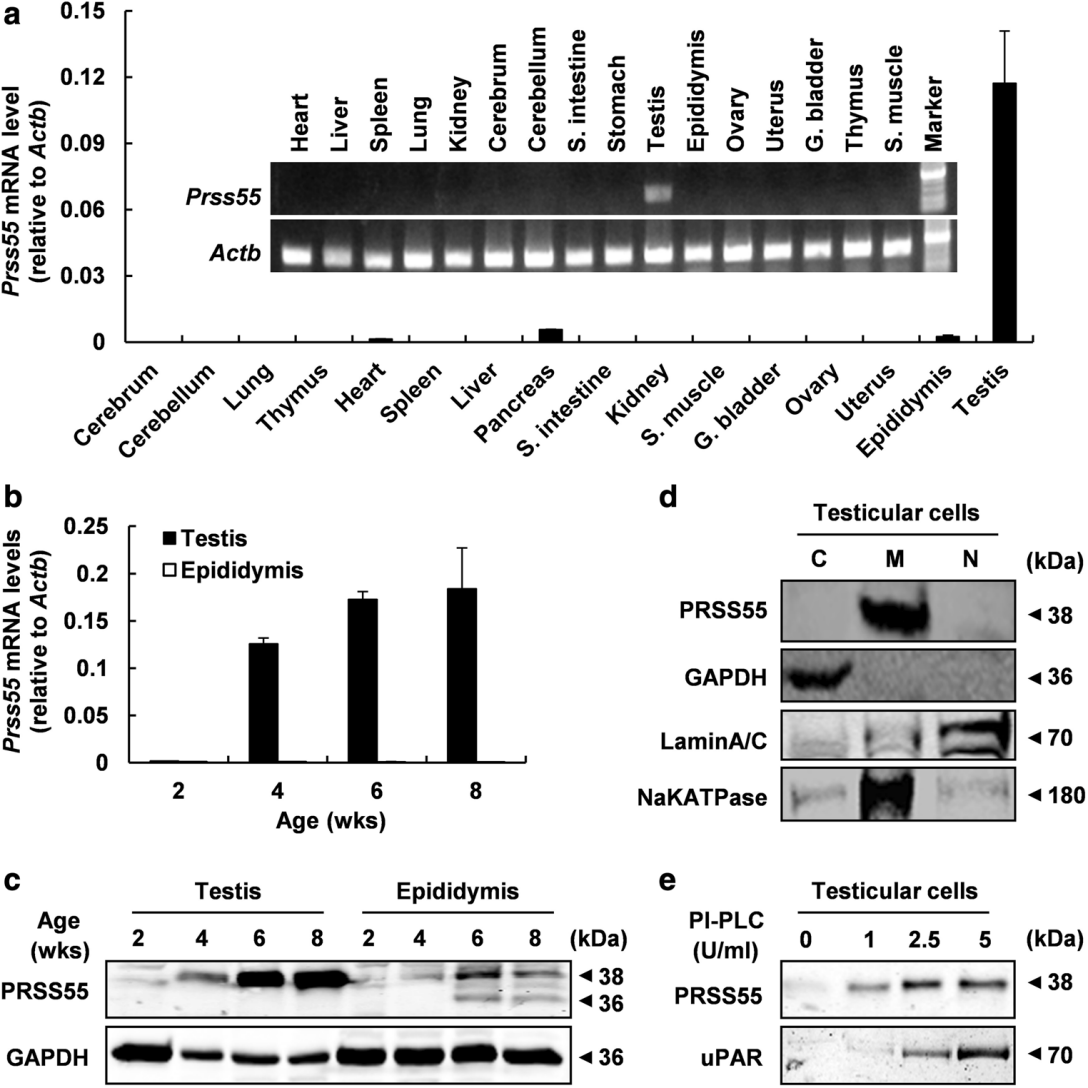
PRSS55 is a GPI-anchored membrane protein specifically expressed in adult mouse testis. **a** Tissue expression profile of *Prss55* mRNA in adult mice is shown by semi-quantitative and real-time quantitative RT-PCR. *Actb* serves as an internal control. Bar chart shows mean ± SE values from three replicates. **b** *Prss55* mRNA relative to *Actb* levels in the testis and epididymis of the mice at the indicated age. Bar chart shows mean ± SE values from three replicates. **c** PRSS55 protein levels in the testis and epididymis of the mice at the indicated age, and GAPDH as a loading control are shown. **d** The protein levels in cytoplasmic (C), membrane (M) and nuclear (N) subfractions of adult mouse testicular cells were analyzed by Western blot. GAPDH, LAMIN A/C and NaKATPASE represent cytoplasmic, nuclear and membrane fractions, respectively. **e** The intact testicular cells from adult mice were treated with the indicated concentrations of PI-PLC, and the supernatants were collected for Western blot analysis. uPAR was detected as a positive control for GPI-anchored protein

The tissue expression profile of *Prss55* in the mouse was examined by both semi-quantitative and real-time RT-PCR. cDNAs were prepared from 16 mouse tissues including cerebrum, cerebellum, lung, thymus, heart, spleen, liver, pancreas, small intestine, kidney, skeletal muscle, gall bladder, ovary, uterus, epididymis, and testis. A unique transcript of mouse *Prss55* was detected exclusively and dominantly in testis (Fig. 1a). The appearance of *Prss55* mRNA and protein in the testis synchronized with the testicular maturation, being detected around 4 weeks old or later (Fig. 1b, c). PRSS55 protein could also be found in the epididymis but different from that in testis, presented as three protein bands with different molecular sizes (Fig. 1c), suggesting a possibility that PRSS55 protein may be modified or cleaved in epididymis. Western blot analysis of the proteins from different fractions of testicular cells reveals that PRSS55 exclusively presented in the membrane fraction (Fig. 1d). Moreover, PRSS55 was GPI-anchored in the testicular cells, since it could be released by PI-PLC treatment, just like uPAR, a known GPI-anchored protein in testis (Fig. 1e).

The subcellular localization of PRSS55 as a GPI-anchored membrane protein was further supported by the evidences from in vitro studies. *pEGFP*-*Prss55* vector was constructed to express a PRSS55-EGFP fusion protein in which EGFP was fused at the C-terminus of the full-length PRSS55 protein. After transient transfection into HEK293 cells, EGFP signals from the cells transfected with *pEGFP*-*Prss55* construct were mainly localized on the cell membrane, in contrast to the EGFP protein alone (Supplementary Fig. S2a). Total lysates of transfected cells were subjected to western blot analysis using anti-PRSS55 antiserum. Two specific protein bands were detected: one was approximately 63 kDa, corresponding to the molecular size of PRSS55-EGFP fusion protein; the other was approximately 38 kDa, probably a GPI-anchored protein (Supplementary Fig. S2b), since it is also released into supernatant by PI-PLC treatment. These data suggest that PRSS55 is a GPI-anchored membrane protein when expressed in HEK293 cells. In addition, PRSS55 in mature sperm could also be released by PI-PLC treatment (Supplementary Fig. S2c).

### Generation of *Prss55*-KO mice

**Fig. 2:**
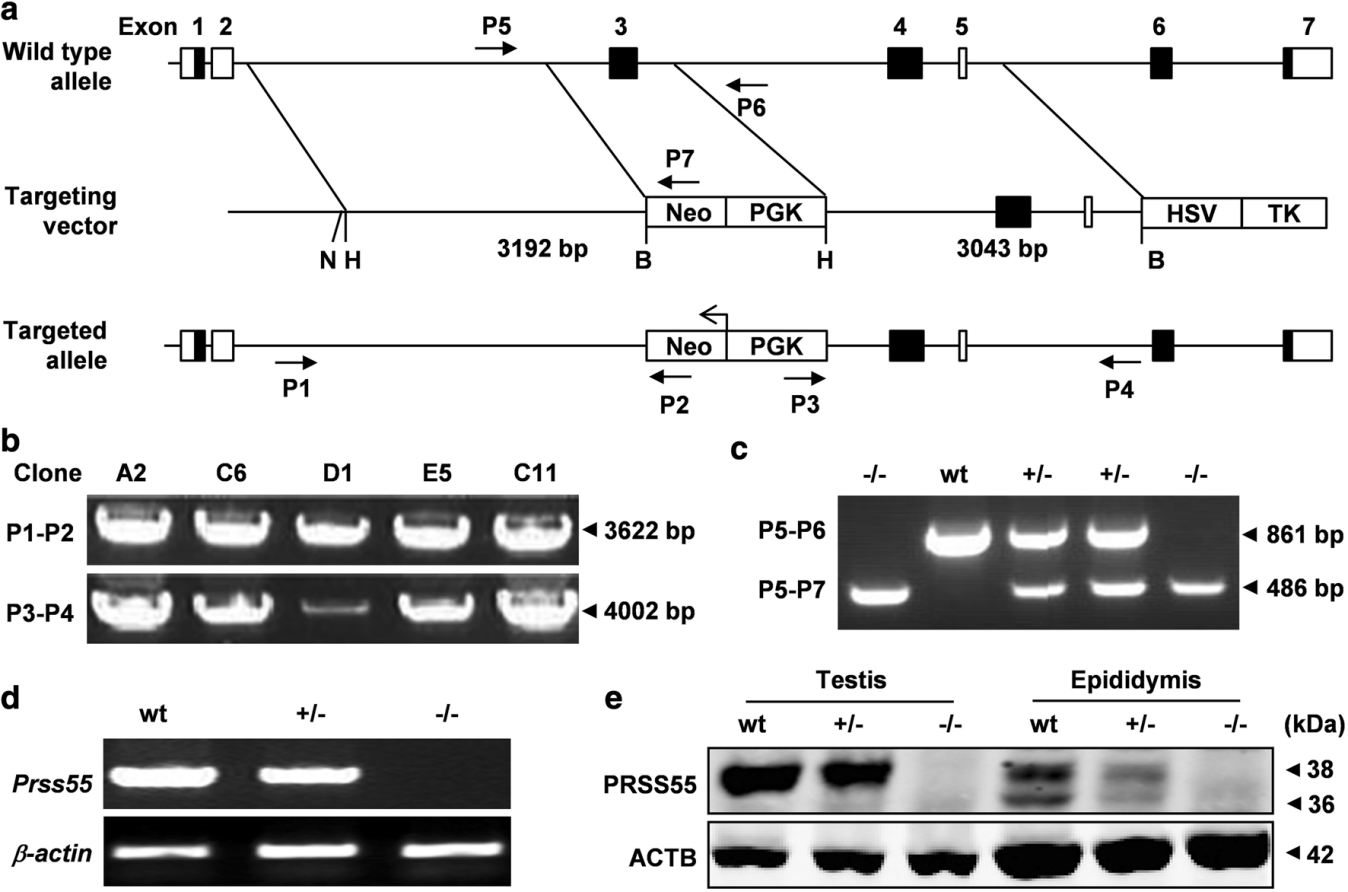
Generation of *Prss55*-KO mice. **a** The targeting strategy for disruption of the mouse *Prss55* gene. The boxes in black represent the exons with coding region. The targeting vector contains 3192 bp of 5′ and 3043 bp of 3′ homologous fragments. PGK-Neo and HSV-TK were used for positive and negative selections, respectively. P1-P7, the primers for genotyping and their relative positions, are indicated. N, *Not*I; H, *Hind*III; B, *Bam*HI. **b** PCR on genomic DNA from ES cell clones was performed using the primers P1 and P2 for the 5′ arm and P3 and P4 for the 3′ arm. **c** Triple primer PCR on mouse tail genomic DNA was performed using primers P5-P7 as routine genotyping of mice. The expected size of PCR products (arrowheads) is shown on the right of the images. **d** RT-PCR for the expression of *Prss55* mRNA in the testis of adult mice with three different genotypes. *Actb* was used as an internal control. **e** Western blot analysis of PRSS55 protein in the testis and epididymis of adult mice with three different genotypes. ACTB was used as a loading control

To explore the physiological function of PRSS55 in vivo, *Prss55*-KO mice were generated using a standard homologous recombination strategy (Fig. 2a). The homologous recombination in ES cells and the three genotypes of the offspring were verified by PCR analysis of genomic DNA (Fig. 2b, c). The absence of PRSS55 expression was confirmed at both RNA and protein levels by semi-quantitative RT-PCR (Fig. 2d), Western blotting (Fig. 2e) and IF analysis (Supplementary Fig. S3).

### Male infertility and impaired in vivo sperm migration in the female genital tract caused by *Prss55* disruption

We first assessed the fertility of *Prss55*-KO mice to determine whether PRSS55 was involved in the regulation of reproduction. Intercrossing of heterozygous mice produced the offsprings following Mendelian inheritance and showing grossly normal development. However, intercrossing of *Prss55*-null males with wt females did not produce any offspring (Table 1), despite normality in plug formation (FCP = 52.6%). In parallel, mating of wt or *Prss55*^+*/*−^ males with wt females during the same period produced 106 and 92 offspring, respectively. The FCP of the wt and *Prss55*^+*/*−^ males was 66.7 and 71.4%, and the FC was 100 and 80%, respectively. On the other hand, the fertility of *Prss55*^−/−^ females was not affected when they were mated with wt or *Prss55*^+/−^ males. The breeding records indicate that 31 heterozygous mice were produced by four *Prss55*^−*/*−^ females mated with wt male mice and 63 mice (33 heterozygous and 30 homozygous mice) were produced by eight *Prss55*^−*/*−^ females mated with *Prss55*^+*/*−^ males. The average litter size of *Prss55*^−*/*−^ females mated with wt and *Prss55*^+*/*−^ males was 7.75 and 7.88, respectively. Together, these data indicate that *Prss55* deficiency in mice leads to male infertility but not female infertility, consistent with the testis-specific expression pattern of PRSS55.

**Table 1:** Targeted disruption of *Prss55* leads to male infertility in mice

Male mice	Females	Plugs	Litters	Offsprings (M/F)	FCP (%)	FC (%)
wt (*n* = 5)	21	14	14	106 (61/45)	66.7	100
*Prss55* ^+ */*−^ (*n* = 5)	21	15	12	92 (48/24)	71.4	80
*Prss55* ^− */*−^ (*n* = 6)	19	10	0	0	52.6	0

FCP = (plugs/females) × 100%; FC = (litters/plugs) × 100%

*FCP* frequency of copulatory plug, *FC* frequency of conception, *M* male, *F* female

**Fig. 3:**
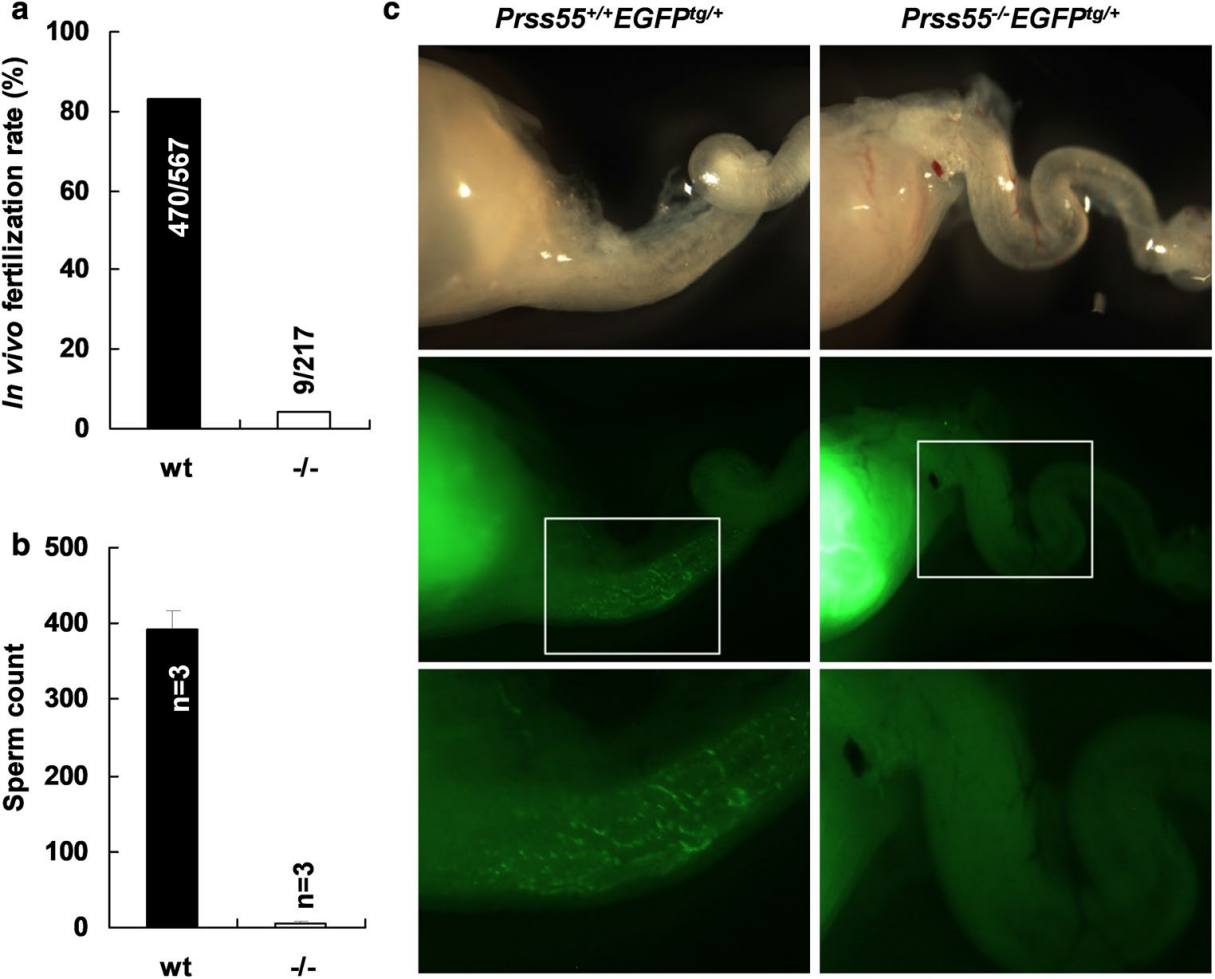
*Prss55*-deficiency dramatically reduces fertilization rate and the defect in sperm migration into oviduct in vivo. **a** wt and *Prss55*^−*/*−^ male mice were mated with superovulated wt females and oocytes were recovered from plugged females. The numbers marked in or above each column represent the total 2-cell embryos/total oocytes obtained from several experiments. **b** Sperm count in the oviduct 3.5 h after coitus. The oviducts of three plugged females each group were dissected and the sperm in the oviducts were flushed out and counted under light microscope. **c** EGFP-expressing wt (*Prss55*^+*/*+^*EGFP*^*tg/*+^) and *Prss55*^−*/*−^ (*Prss55*^−*/*−^*EGFP*^*tg/*+^) males were mated with wt females. The uterus and oviduct were visualized under fluorescence microscope 3.5 h after coitus. Boxed areas in the middle images are enlarged as the bottom ones

To further confirm the phenotype of male infertility of *Prss55*-KO mice, we performed the analysis of in vivo fertilization rate. The wt and *Prss55*^−*/*−^ males were mated with superovulated wt females and the fertilized oocytes were recovered from the oviducts 45 h after hCG injection. The in vivo fertilization rate calculated as the percentage of the 2-cell embryos was 82.9% in wt mice and 4.1% in mutant mice (Fig. 3a), suggesting that the infertility of *Prss55*^−*/*−^ males was mainly due to the fertilization defects but not due to the developmental disability of the embryos.

To address the underlying causes of the dramatically decreased in vivo fertilization rate of *Prss55*^−*/*−^ males and thus male infertility, we evaluated the in vivo sperm migration in the female genital tract by counting sperm present in the oviduct 3.5 h after coitus and by tracing the migration of EGFP-expressing sperm from uterus into oviduct. As shown in Fig. 3b, more than 300 wt sperm were recovered from the oviduct of females mated with wt males, while less than 10 *Prss55*^−*/*−^ sperm were recovered under the same experimental procedures. The EGFP-expressing sperm with wt *Prss55* alleles were obtained from pAcr-SP-NTP-EGFP transgenic mice [27] and the EGFP-expressing sperm with *Prss55*-KO alleles were obtained from mice generated by crossing *Prss55*^−*/*−^ females with pAcr-SP-NTP-EGFP transgenic males followed by mating between siblings. In vivo tracing of the EGFP-expressing *Prss55*^−*/*−^ sperm (*Prss55*^−*/*−^*EGFP*^*tg/*+^) showed that *Prss55*^−*/*−^ sperm were mainly confined in the uterus after they were ejaculated and were hardly able to enter the oviduct, in contrast to the distribution of the EGFP-expressing wt sperm (*Prss55*^+*/*+^*EGFP*^*tg/*+^) in the oviduct (Fig. 3c). Both these data demonstrate that sperm from *Prss55*^−*/*−^ males are impaired in migration from uterus into oviduct in vivo.

### *Prss55* disruption results in defective sperm–egg binding but normal fertilization rate in vitro

To understand the underlying causes of male infertility of *Prss55*^−*/*−^ mice, we first performed pathological analyses of testis and epididymis tissues from mice of three different genotypes. H&E staining reveals that sperm are almost normally produced in the testis and transported through the epididymis (Supplementary Fig. S4a). And there is no significant difference in the ratio of testis/body weight between wt and *Prss55*^−*/*−^ mice (Supplementary Fig. S4b, *p* = 0.62, *n* = 5 each group). The morphology of sperm evaluated by H&E staining and TEM suggested that the gross morphology and the ultrastructures of *Prss55*^−*/*−^ sperm were similar to those of wt sperm (Supplementary Fig. S4c). Moreover, we assessed the motility of sperm isolated from the cauda epididymis by CASA and no significant differences in the movement characteristics were identified between wt and *Prss55*^−*/*−^ sperm (Supplementary Table S2). The sperm acrosome is a sac-like structure surrounded by inner and outer acrosomal membranes. AR causes the exocytosis of sperm acrosomal contents, especially enzymes that facilitate sperm to penetrate the ZP and fertilize the egg [32]. Thus, the inducibility of AR is an important indicator for evaluating the fertilizing capability of sperm. Here, we used a calcium ionophore, A23187, to induce AR and evaluated the status of acrosome by PNA staining. The results show that the inducibility of AR is comparable between wt and *Prss55*^−*/*−^ sperm (Supplementary Fig. S4d). Moreover, the activity of acrosomal enzymes released by the induction of AR was examined by four different substrates. There were also no significant differences existed between wt and *Prss55*^−*/*−^ sperm (Supplementary Fig. S4e-h). All these above-mentioned data suggest that the infertility of *Prss55*^−*/*−^ males is probably irrelevant to the sperm production, sperm morphology, sperm motility, AR, and the activity of acrosomal enzymes.

**Fig. 4:**
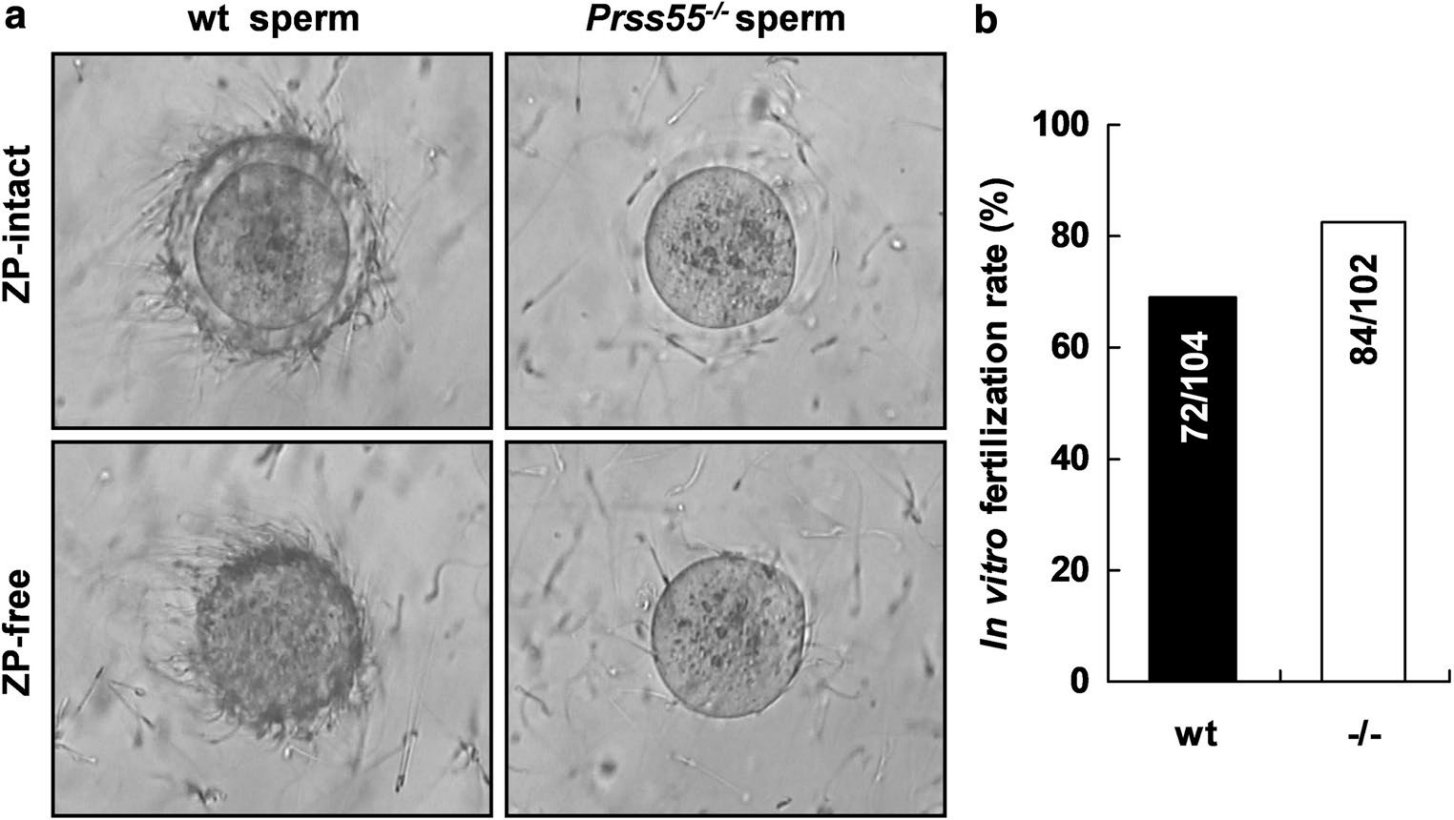
*Prss55* disruption results in defective sperm–egg binding but normal fertilization rate in vitro. **a** Zona-intact and zona-free eggs were incubated with capacitated wt or *Prss55*^−*/*−^ sperm in vitro. *Prss55*^−*/*−^ sperm show severe defects in recognition/binding to either zona-intact or zona-free oocytes. One representative experiment out of three is shown. **b** IVF rate is compared between wt and *Prss55*^−*/*−^ sperm. The numbers indicated in each column are the total oocytes developed to 2-cell stage and the total oocytes incubated with sperm at the beginning of the fertilization assays from several experiments

Next, we performed the IVF assay to see whether the fertility of the *Prss55*^−*/*−^ sperm in vitro was disturbed or not. Notably, we observed severe defects of *Prss55*^−*/*−^ sperm in recognition/binding to either zona-intact or zona-free oocytes in vitro, compared to the wt controls (Fig. 4a). In contrast to the dramatically lower in vivo fertilization rate of *Prss55*^−*/*−^ males (Fig. 3a), the IVF rate of *Prss55*^−*/*−^ sperm was comparable to that of wt sperm (Fig. 4b). These findings suggest that *Prss55* deficiency in mice impairs not only the in vivo migration ability but also the in vitro sperm–egg recognition/binding of the sperm.

To clarify how PRSS55 functions in sperm–egg recognition/binding, we further investigated the subcellular localization of PRSS55 in mature sperm. PRSS55 was detected at the acrosomal region by IF analysis. PNA, which binds to the outer acrosomal membrane of non-acrosome-reacted sperm, was used as a probe to indicate the acrosomal region of sperm (Supplementary Fig. S5). In addition, unlike the soluble acrosomal protein Acr-EGFP, PRSS55 could not be completely released by A23187-induced AR. ADAM2 and Acr-EGFP represent typical cell surface and soluble acrosomal proteins, respectively, and they served as controls (Supplementary Fig. S6a). When the sperm suspension was treated with different concentrations of proteinase K, PRSS55 could be released into supernatant, with a similar pattern to that of the membrane protein ADAM2, whereas the intracellular protein Acr-EGFP remained inside the sperm (Supplementary Fig. S6b). These data indicate that PRSS55 is a sperm-surface membrane protein in mature sperm. Proteinase K digestion of testicular cells also suggests that PRSS55 is a cell-surface protein (Supplementary Fig. S6c). However, anti-PRSS55 serum was unable to block the binding of wt sperm with either zona-intact or zona-free oocytes (Supplementary Fig. S7), suggesting that PRSS55 may be indirectly involved in sperm–egg binding.

### *Prss55* deficiency impedes ADAM3 maturation and differentially expressed genes are identified by microarray analysis

**Fig. 5:**
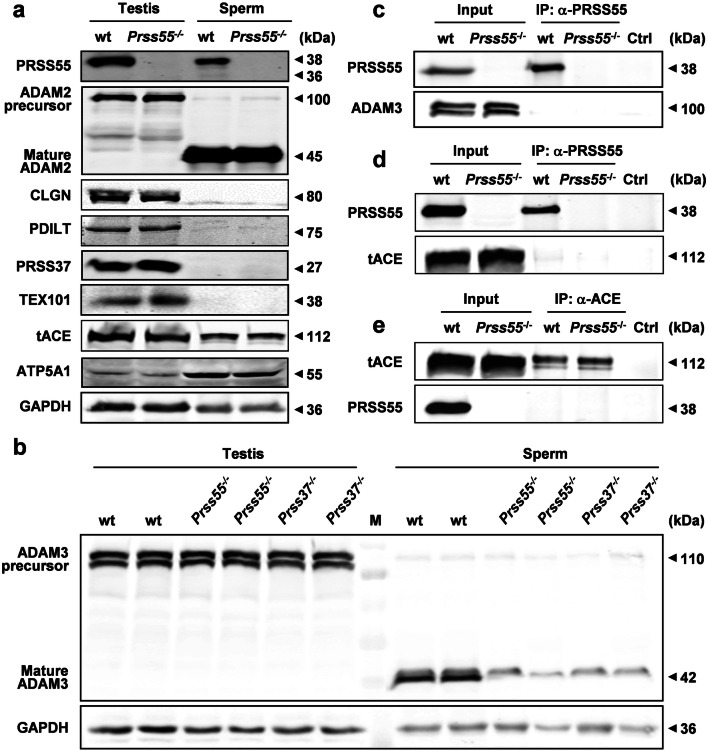
*Prss55* deficiency impedes ADAM3 maturation with no effects on the related proteins as marked. **a** The total lysates of testis and sperm were subjected to immunoblot analysis using the indicated antibodies. No significant differences between wt and mutant mice in protein levels are shown. ATP5A1 and GAPDH are used as loading controls. **b** Western blot analysis of ADAM3 precursor and mature ADAM3 in the testis and sperm of wt, *Prss55*^−*/*−^ and *Prss37*^−*/*−^ mice. GAPDH is used as a loading control. **c**, **d**, **e** Testicular cell extracts from wt and *Prss55*^−*/*−^ mice were immunoprecipitated and probed with antibodies against ADAM3, PRSS55, and tACE. Testicular cells lysates were loaded as the input control. No evidence for the interaction of PRSS55 with ADAM3 and tACE was found

The defects of in vivo migration from uterus into oviduct and in vitro sperm–egg recognition/binding discovered in the *Prss55*^−*/*−^ sperm prompted us to examine the expression level of mature ADAM3 in the cauda epididymal *Prss55*^−*/*−^ sperm, since many gene knockout mice sharing these phenotypes showed impaired maturation or aberrant distribution of mature ADAM3 in sperm. As illustrated in Fig. 5b, mature ADAM3 is nearly absent from the *Prss55*^−*/*−^ sperm, while its precursor form expressed in the testis is almost unaffected. We also examined the expression level of ADAM2, a closely related ADAM family member to ADAM3, and found that its maturation was not affected by *Prss55* deficiency (Fig. 5a). In addition, the protein levels of CLGN, PDILT, TEX101, tACE, and PRSS37 were also found to be normal in the testes of *Prss55*^−*/*−^ males (Fig. 5a). Moreover, no evidence for the interaction of PRSS55 with ADAM3 and tACE was found (Fig. 5c–e) in the mouse testis and PRSS55 was not a substrate of tACE (Supplementary Fig. S8b). Since *Prss37*-KO mice show similar phenotype with *Prss55*^−*/*−^ mice [16], we further examined the expression of PRSS55 in the testis and sperm of *Prss37*^−*/*−^ males to see whether PRSS37 functioned upstream of PRSS55. The results showed that PRSS55 protein levels were not significantly changed by *Prss37* deficiency (Supplementary Fig. S8a, c). These data suggest that *Prss55* deficiency in mice affects the maturation of ADAM3 in sperm and the disappearance of mature ADAM3 in *Prss55*^−*/*−^ sperm is not caused by abnormal expression of ADAM2, CLGN, PDILT, TEX101, tACE, and PRSS37, although the involvement of other proteins in this process and their relationship with PRSS55 remain to be determined.

**Fig. 6:**
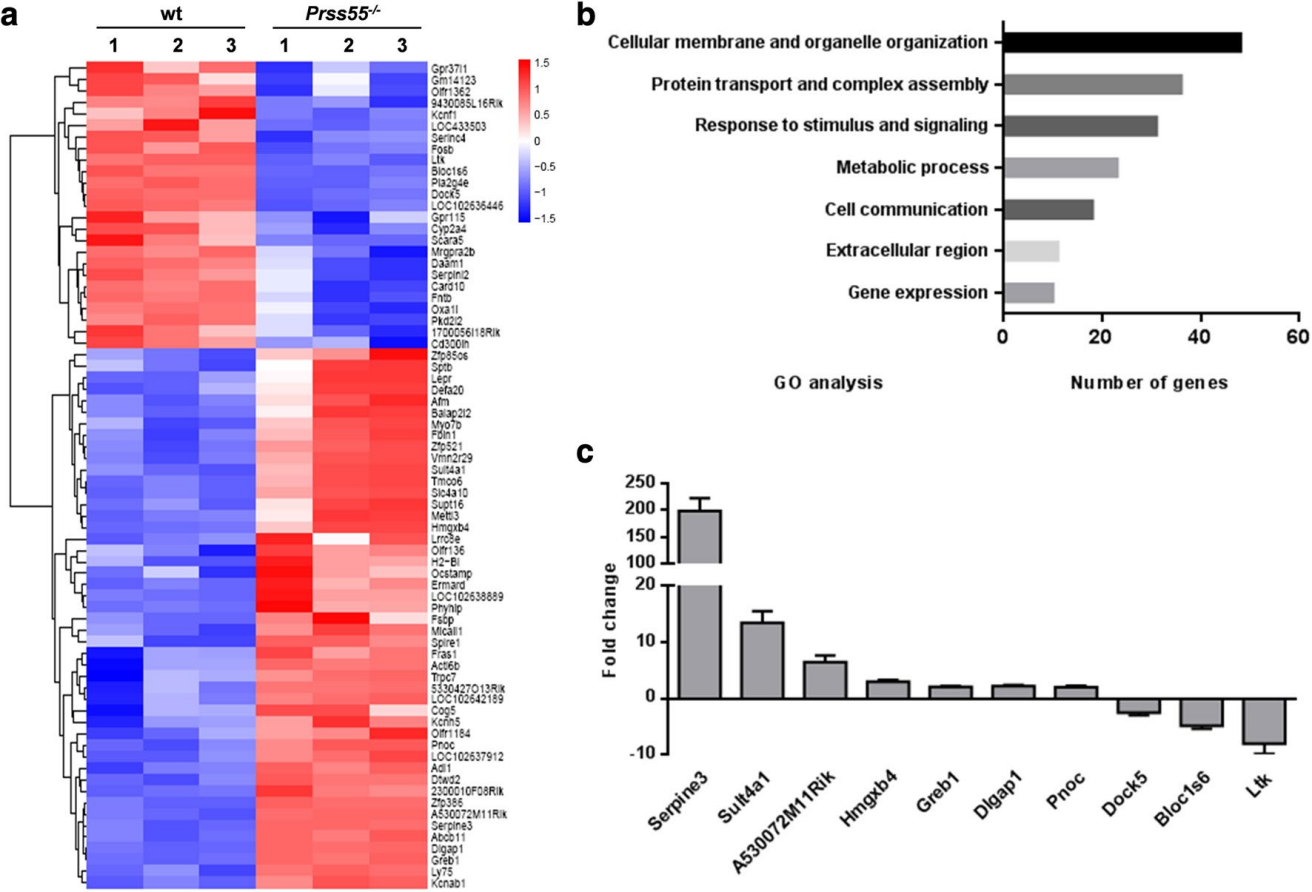
Differentially expressed genes in the testis of *Prss55*^−*/*−^ mice identified by microarray analysis. **a** Total RNA extracted from the testes of three pairs of wt and *Prss55*^−*/*−^ mice were used for gene expression analysis using whole mouse genome 4 × 44 k microarray (Agilent Technologies). A total of 72 genes were identified to be differentially expressed significantly (*p* < 0.05, *f*_c_ ≥ 2), which are displayed as the heat map. **b** GO analysis of the 72 DE genes, which primarily clustered into 7 functional groups with varied numbers. **c** Fold changes of ten genes (seven up-regulated and three down-regulated genes) were examined by qRT-PCR. The housekeeping gene *Actb* was used for expression normalization. Bar chart shows mean ± SE values of six mice

Microarray analysis of total testis RNAs from wt and *Prss55*^−*/*−^ mice (*n* = 3 each group) was performed to identify DE genes (*p* < 0.05, *f*_c_ ≥ 2) caused by *Prss55* deficiency. The volcano plot of the microarray data was shown in Supplementary Fig. S9. Results showed that a total of only 72 genes were disregulated in the testis of *Prss55*^−*/*−^ mice, with 47 genes up-regulated and 25 genes down-regulated (Fig. 6a), after eliminating those designated by NA and repeats detected by more than one probe. The information of these DE genes was listed in Supplementary Table S3. Gene ontology (GO) analysis of these DE genes suggested that most of these genes were associated with cellular membrane and organelle organization (48 genes), protein transport and complex assembly (36 genes), and response to stimulus and signaling (31 genes) (Fig. 6b), consistent with the dramatic structural changes and dynamic protein modifications in the spermatids during the highly regulated process of spermiogenesis. We used qRT-PCR to validate the microarray results. The fold changes of ten genes (seven up-regulated genes and three down-regulated genes) examined by qRT-PCR are illustrated in Fig. 6c and were compared with the fold change data from the microarray analysis (Supplementary Table S4). Although the fold change values were not match exactly due to variations in the detection technology, the changing trends were basically consistent. Among the significantly regulated genes validated by qRT-PCR, Serpine3, Sult4a1 and A530072M11Rik were highly up-regulated while Bloc1s6 and Ltk were highly down-regulated. The fold changes of these genes were 198.85, 13.45, 6.55, − 4.82 and − 8.01, respectively. However, the mechanisms by which the disruption of *Prss55* affects the expression of these genes and how they play in the male fertility remain to be investigated.

## Discussion

In the present study, we have demonstrated that *Prss55*, a member of the serine protease family, is a GPI-anchored membrane protein not only in testicular cells, but also in cultured cells and sperm. Its deficiency in mice results in male infertility with impaired sperm migration from uterus into oviduct and defective sperm-zona and sperm–egg recognition/binding. However, the mating activity, sperm production, sperm morphology, sperm motility, inducibility of AR, activity of acrosomal enzymes and in vitro sperm fertility were almost normal compared to those of wt controls. These phenotypes of *Prss55*^−*/*−^ male mice were quite similar to other gene-KO mouse models described previously, including *Prss37*-KO mice. The main difference between PRSS37 and PRSS55 is that PRSS37 is lost during late steps of spermiogenesis, whereas PRSS55 remains present in the mature sperm. However, disruption of each of them does not affect the expression level of the other in testis and sperm (Supplementary Fig. S8a) and even during the epididymal sperm maturation process (Supplementary Fig. S8c), suggesting that they are independent of each other and both of them are upstream of a downstream target(s), such as ADAM3.

ADAM3 undergoes posttranslational modification during the epididymal transit and is located at the acrosomal membranes of the sperm [33]. The maturation process of ADAM3 is very complicated that many genes have been reported to be involved in this process. The impaired maturation of ADAM3 on the sperm membrane is another common feature of the above mentioned gene-KO mice. Up till now, the mechanism by which the mature form of ADAM3 decreases in the sperm of these gene-KO mice remains unclarified. Precursor ADAM3 has been reported to be interacted with CLGN [34], CALR3 [5], and PDILT [13] on the ER membrane, and with ADAM2 [35], ADAM6 [36] and TEX101 [17] on the surface of mouse testicular germ cells. And TEX101 is a substrate of tACE. When TEX101 remained on sperm by ACE disruption, the localization of ADAM3 on sperm became aberrant, and males became infertile probably due to the production of nonfunctional ADAM3 [17, 34]. According to our data, we have excluded the effects of ADAM2, CLGN, PDILT, TEX101, tACE, and PRSS37 on the maturation of ADAM3. And no interaction of PRSS55 with ADAM3 and tACE was detected (Fig. 5c–e) in the mouse testis and PRSS55 was not a substrate of tACE, either (Supplementary Fig. S8b).

We speculate that PRSS55 does not influence the maturation of ADAM3 by direct physical interaction, but rather indirectly through other intermediates. Thus, we carried out microarray analysis of total testis RNAs from wt and *Prss55*^−*/*−^ mice (*n* = 3 each group) to identify DE genes (*p* < 0.05, *f*_c_ ≥ 2) caused by *Prss55* deficiency. These DE genes might play important roles in the maturation process of key factors relating to sperm fertility, including ADAM3. In line with our expectations, GO analysis of these DE genes suggested that most of these genes were associated with cellular membrane and organelle organization, protein transport and complex assembly, and response to stimulus and signaling (Fig. 6b), consistent with the dramatic structural changes and dynamic protein modifications in the spermatids during the highly regulated process of spermiogenesis. Among the 72 DE genes in the testis of *Prss55*^−*/*−^ mice (Fig. 6a) and the 28 DE genes in the testis of *Prss37*^−*/*−^ mice (data not shown), there were seven common genes: Bloc1s6, Cyp2a4, Ltk, Cd3001h, Gpr115, Olfr1362 and Pla2g4e. All of them were down-regulated in the testis of both *Prss55*^−*/*−^ and *Prss37*^−*/*−^ mice. qRT-PCR validation results showed that the fold change of Bloc1s6 was − 4.82 in *Prss55*^−*/*−^ testis and − 5.66 in *Prss37*^−*/*−^ testis, the fold change of Ltk was − 8.01 in *Prss55*^−*/*−^ testis and − 10.79 in *Prss37*^−*/*−^ testis, and the fold change of Gpr115 was − 5.13 in *Prss55*^−*/*−^ testis and − 2.1 in *Prss37*^−*/*−^ testis. However, the fold changes of the other four genes were less than 2 in both Prss55^−/−^ and Prss37^−*/*−^ testis. On the other hand, Serpine3, Sult4a1 and A530072M11Rik were three genes specifically up-regulated in *Prss55*^−*/*−^ but not *Prss37*^−*/*−^ testis. Their fold changes were 198.85, 13.45 and 6.55, respectively. Of course, we did not validate the expression level of all the 93 DE genes by qRT-PCR. Maybe, some of them exhibited similar up- or down-regulation caused by *Prss55* or *Prss37* deficiency. All in all, we believe that there are some common characteristics in gene expression regulation between *Prss55*-KO mice and *Prss37*-KO mice; however, differences still existed.

Through a literature search, we can see that several functions of the above-mentioned DE genes have been studied, but none of these studies involve their functions in the testis or male fertility. Our microarray results suggest that these genes might play important roles in spermatogenesis and/or sperm maturation. The functional network of how these genes are coordinated to regulate this process needs to be further investigated, especially the molecular mechanisms on how PRSS55 assists the presence of mature ADAM3 in sperm.

Although PRSS55 remains in the mature sperm, unlike PRSS37, which was discarded at the end of spermiogenesis, we cannot conclude that the failure of sperm migration in vivo and sperm–egg interaction in vitro caused by *Prss55* deficiency was directly attributed to PRSS55. These defects are more likely caused by the absence of mature ADAM3 in sperm, since it is a common feature shared by many other gene-KO mouse models that display similar defects.

In summary, the testis-expressed and sperm-located PRSS55 protein plays a crucial role in the fertility of male mice. It is a novel gene required for ADAM3 maturation, in vivo sperm migration and in vitro sperm–egg interaction, but not required for the fertilizing capability of sperm in vitro. Its deletion in the testis destroys the normal expression of multiple genes, most of which are associated with cellular membrane and organelle organization, protein transport and complex assembly, and response to stimulus and signaling. Future dissecting the functional network of these genes will facilitate our understanding of the sperm maturation process.

### Electronic supplementary material

Below is the link to the electronic supplementary material.

Supplementary material 1 (DOCX 2940 kb)

## Abbreviations

ZP: Zona pellucida
ER: Endoplasmic reticulum
GPI: Glycosylphosphatidylinositol
UMI: Unexplained male infertility
KO: Knockout
wt: Wild type
PBS/SDS: PBS containing 1% SDS
HCl/SDS: 1 mM HCl containing 1% SDS
IP: Immunoprecipitation
PI-PLC: Phosphatidylinositol-specific phospholipase C
uPAR: Urokinase-type plasminogen activator receptor
AR: Acrosome reaction
PMSF: Phenylmethanesulfonyl fluoride
IF: Immunofluorescence
H&E: Hematoxylin and eosin
TEM: Transmission electronic microscopy
PNA: Peanut agglutinin
FCP: Frequency of copulatory plug
FC: Frequency of conception
PMSG: Pregnant mare serum gonadotrophin
hCG: Human chorionic gonadotropin
CASA: Computer-assisted semen analysis
MUP: 4-Methylumbelliferyl phosphate
Boc: *t*-Butyloxycarbonyl
MCA: 4-Methylcoumaryl-7-amide
AGPR: Boc-Ala-Gly-Pro-Arg-MCA
VPR: Boc-Val-Pro-Arg-MCA
LTR: Boc-Leu-Thr-Arg-MCA
OCCs: Oocyte-cumulus complexes
IVF: In vitro fertilization
HTF: Human tube fluid
KSOM: Potassium simplex medium
DE: Differentially expressed
GEO: Gene expression omnibus
NCBI: National center for biotechnology information
SE: Standard error
GO: Gene ontology
