# Supplementary material for: Serine protease PRSS55 is crucial for male mouse fertility via affecting sperm migration and sperm–egg binding
Source: Cell Mol Life Sci. 2018 Jul 21;75(23):4371–84. doi: 10.1007/s00018-018-2878-9 (PMC6208766; doi:10.1007/s00018-018-2878-9)
Supplement: Supplementary file 1 — Supplementary material 1 (DOCX 2940 kb) [file 18_2018_2878_MOESM1_ESM.docx]

**SUPPORTING INFORMATION**


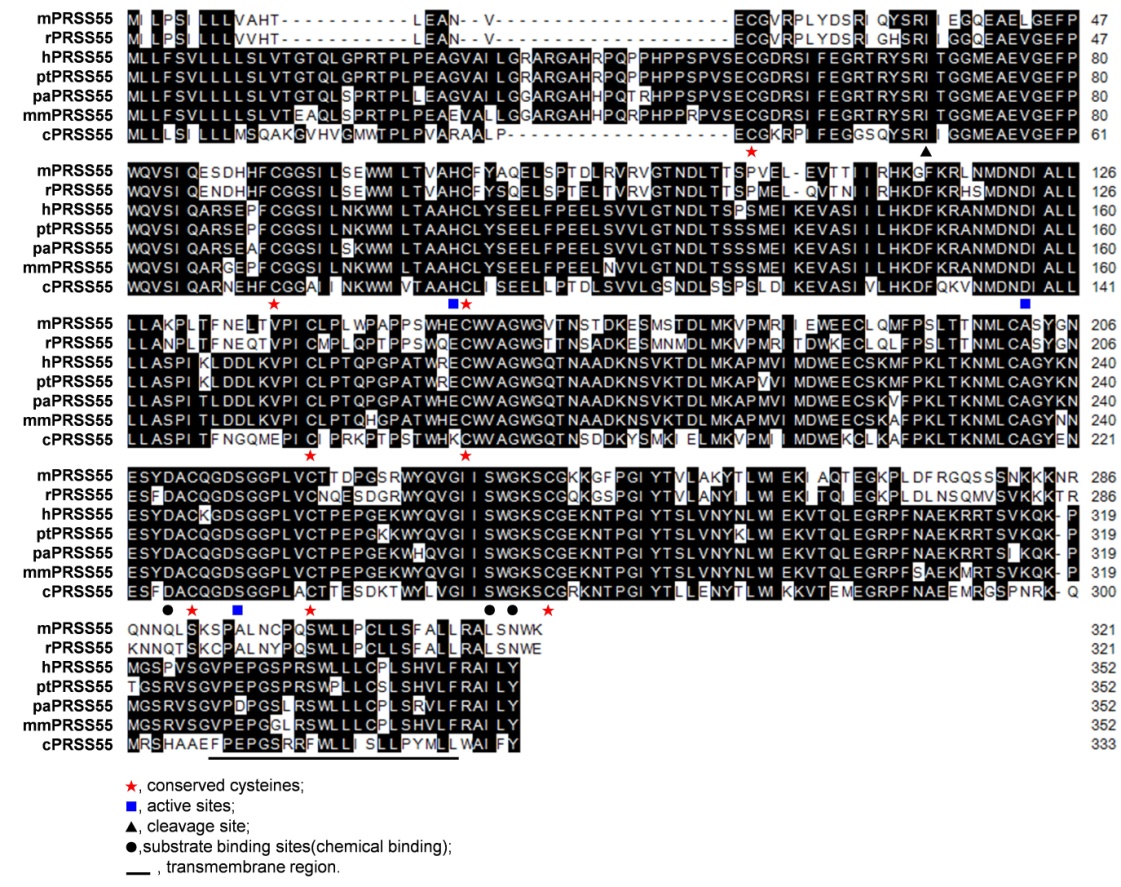


**Fig. S1** PRSS55 shares highly conserved amino acids among species. Comparison of amino acid sequences of PRSS55 from seven different mammalian species using the program Clustal W. Consensus amino acids are shaded in black. Red stars indicate conserved Cys residues, blue squares are active sites, black triangle is a putative cleavage site, black solid circles are substrate binding sites and black underline shows transmembrane region. The amino acid sequences of these proteins were extracted from the GenBank^TM^ database. The accession numbers are as follows: mPRSS55 (NP_001074532), rPRSS55 (XP_008767522), hPRSS55 (NP_940866), ptPRSS55 (XP_528278), paPRSS55 (XP_002818861), mmPRSS55 (XP_001089204), cPRSS55 (XP_013962962). m, *Mus musculus* (house mouse); r, *Rattus norvegicus* (Norway rat); h, *Homo sapiens* (human); pt, *Pan troglodytes* (chimpanzee); pa, *Pongo abelii* (Sumatran orangutan); mm, *Macaca mulatta* (Rhesus monkey); c, *Canis lupus familiaris* (dog).


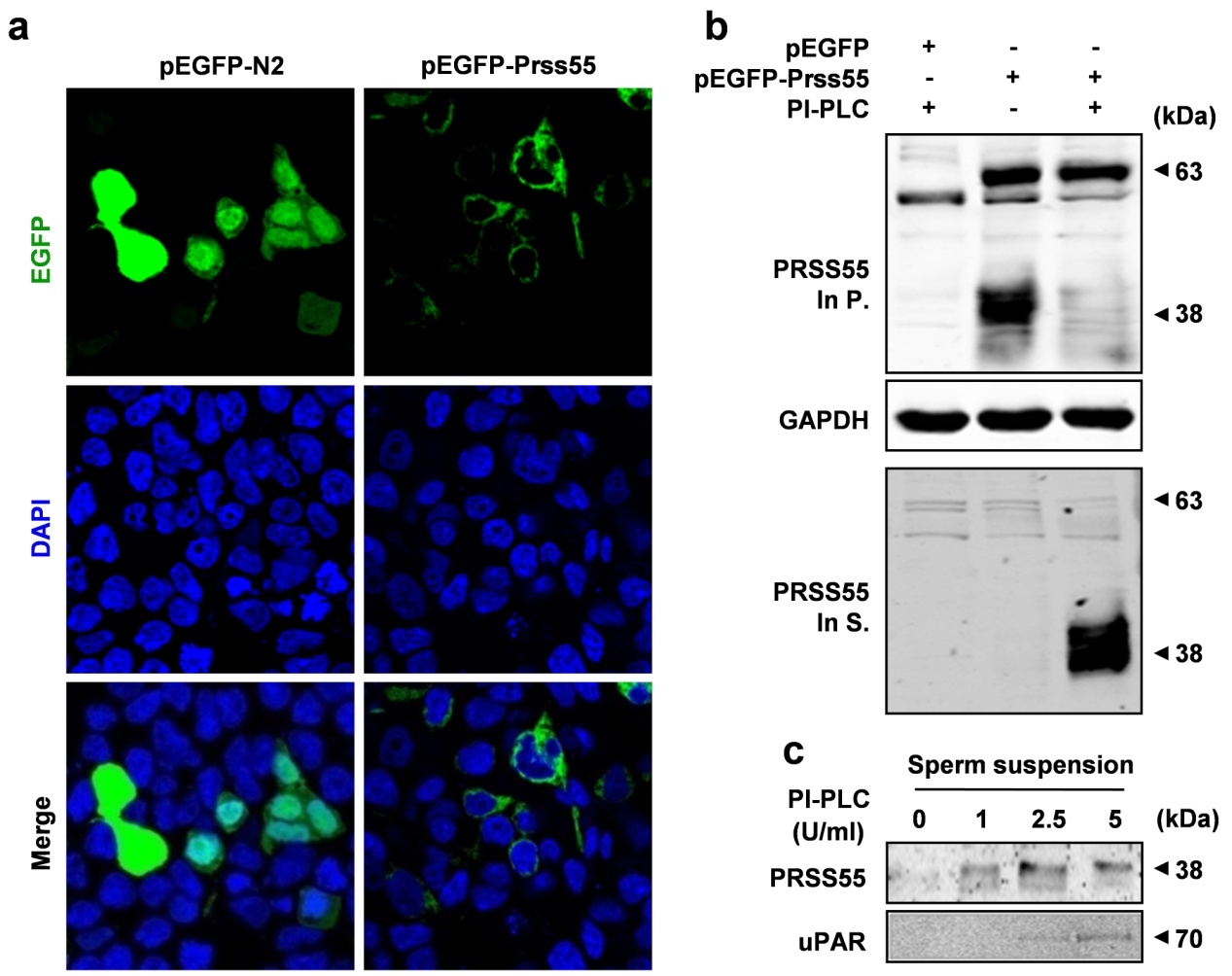


**Fig. S2** PRSS55 is a GPI-anchored membrane protein. **a** HEK293 cells transfected with pEGFP-N2 or pEGFP-Prss55 expression vector for 48 h were subjected to confocal microscopy. **b** HEK293 cells transfected with pEGFP or pEGFP-Prss55 for 48 h were treated with/without 2.5 U/ml PI-PLC for 1 h at 37°C. The protein levels of PRSS55 in supernatants and in pellets were immunodetected by Western blotting. **c** Equal aliquots of sperm suspension in PBS were treated with various concentrations (0, 1, 2.5, 5 U/ml) of PI-PLC for 1 h at 37°C. PRSS55 in the supernatants was analyzed by Western blotting. uPAR was detected as a positive control for GPI-anchored protein.


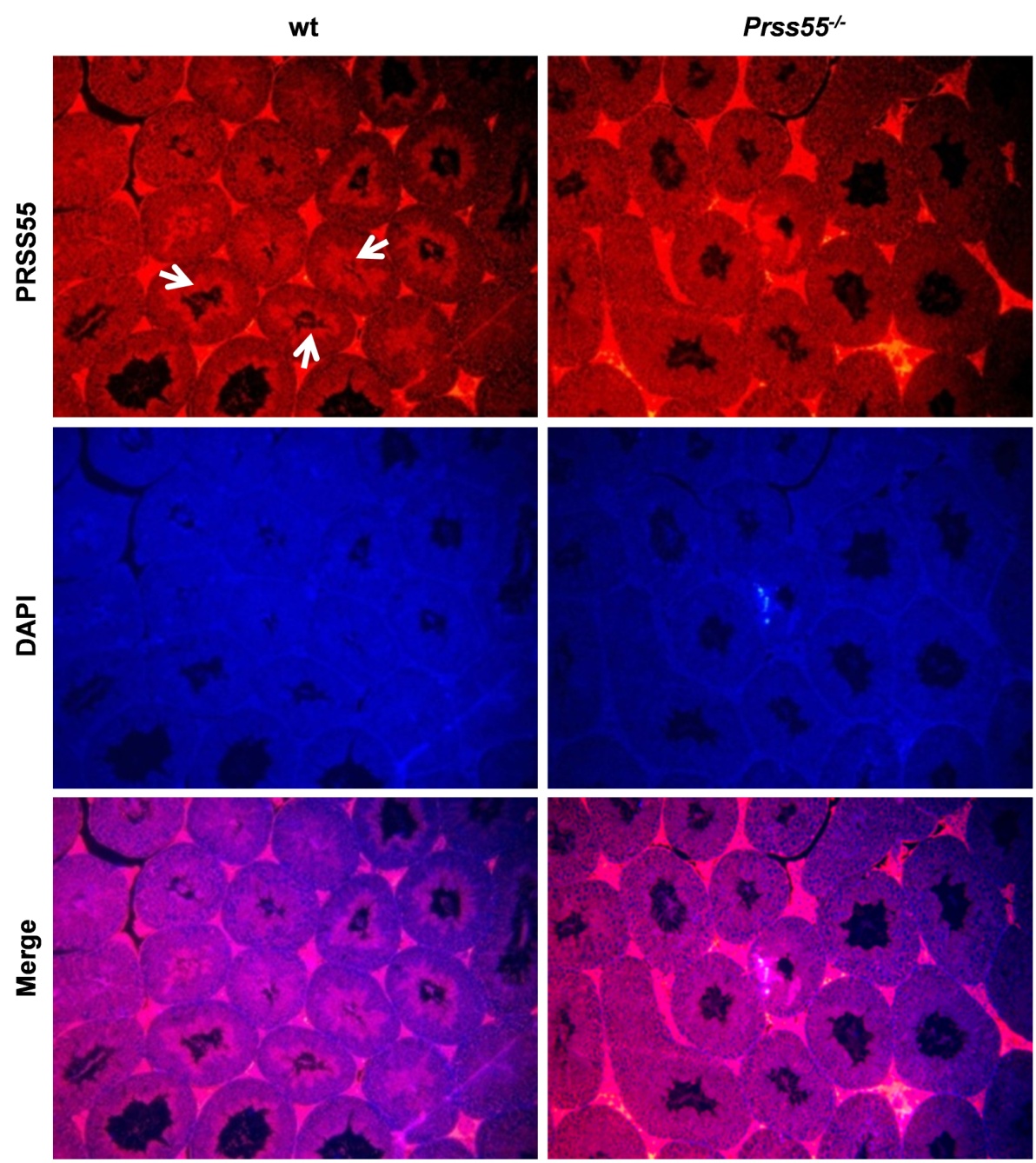


**Fig. S3** Distribution of PRSS55 protein in the adult mouse testis was assayed by immunofluorescence. White arrows point to specific staining of PRSS55 protein, mainly at the luminal side of the [seminiferous](javascript:;) [tubule](javascript:;)s. Nuclei were visualized by DAPI staining.


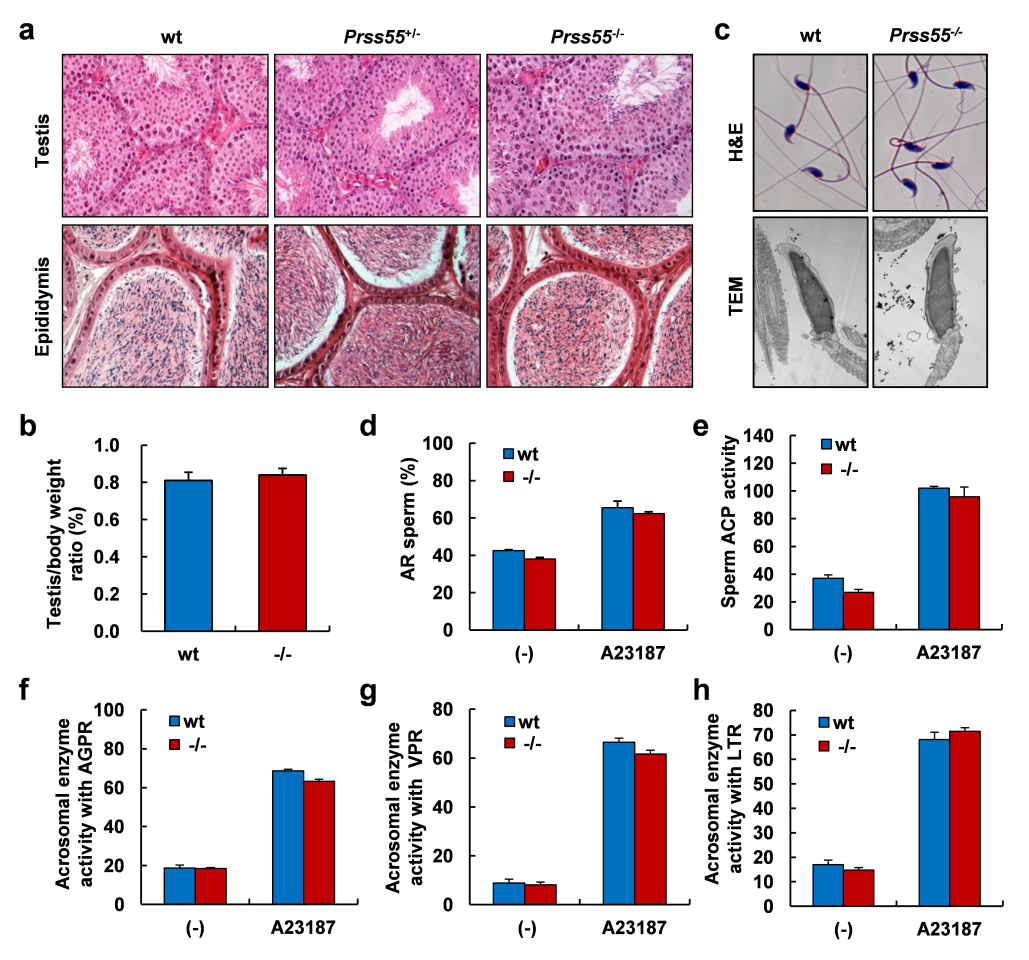


**Fig. S4** Targeted deletion of *Prss55* has no effects sperm production, sperm morphology, acrosome reaction, and the activity of acrosomal enzymes. **a** H&E staining images of testis and cauda epididymis from mice of three different genotypes. **b** Testis size of wt and *Prss55^-/-^* mice evaluated by the ratio of testis weight to body weight (n = 5 for each group). **c** Morphology of cauda epididymal sperm evaluated by H&E staining and TEM. **d** Induction of acrosome reaction by A23187 was comparable between wt and *Prss55^-/-^* sperm. The status of acrosome was examined by PNA staining after spotting sperm onto glass slides. **e-h** The activity of acrosomal enzymes released by the induction of acrosome reaction was measured by using four different substrates. The activity of acrosomal enzymes was expressed as the fluorescence intensity. Error bars indicate the standard error of the mean data from three mice. No significant differences existed between wt and *Prss55^-/-^* sperm.


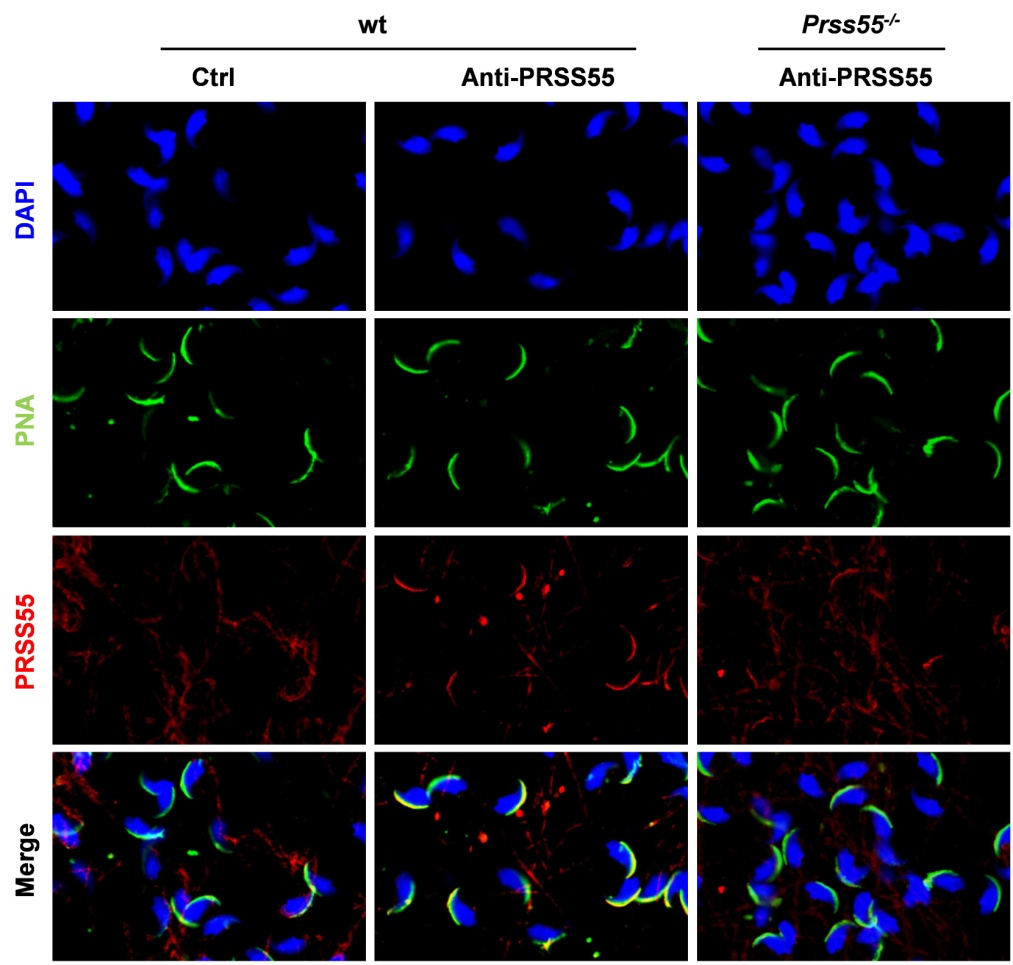


**Fig. S5** PRSS55 is localized in acrosome region of mature sperm. Sperm from cauda epididymal of wt and *Prss55*^-/-^ mice were immunostained using anti-PRSS55 antibody (red) or pre-immune serum. The acrosome and nucleus were also stained with Alexa Fluor 488-conjugated peanut lectin PNA (green) and DAPI (blue), respectively.


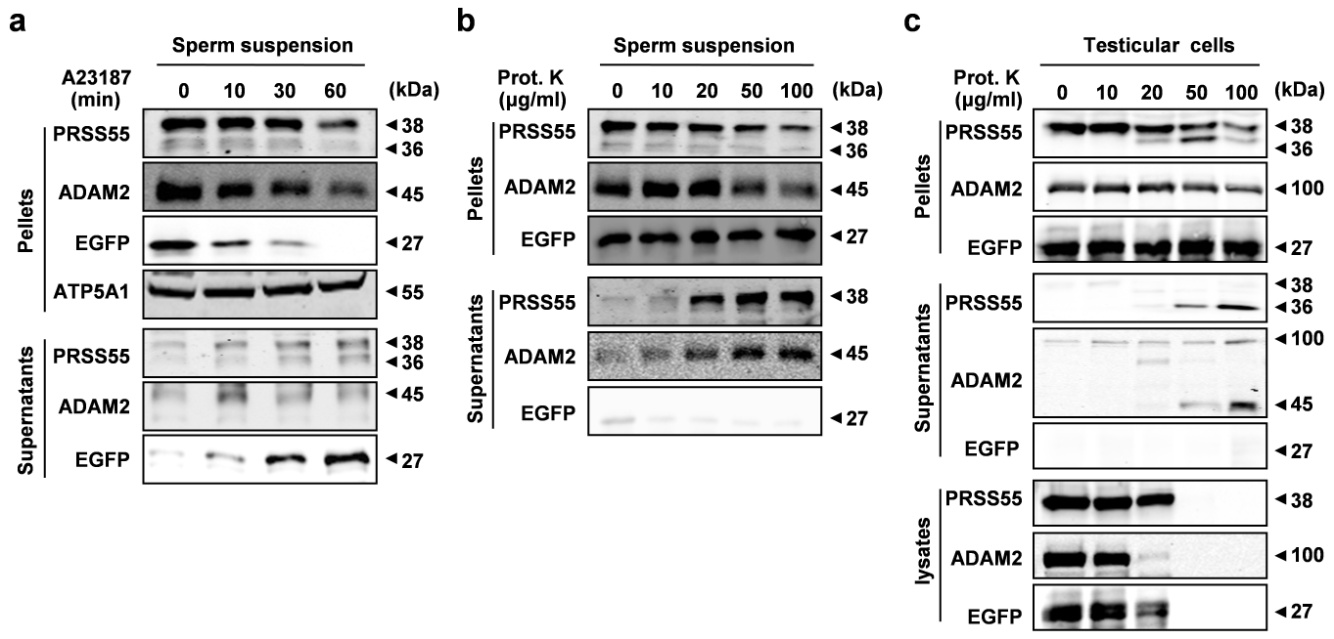


**Fig. S6** Release kinetics and subcellular localization of PRSS55 protein in germ cells. **a** Sperm suspension of pAcr-SP-NTP-EGFP transgenic mice were treated with A23187 for the indicated time. The supernatants and pellets were analyzed by Western blotting for the proteins as marked. Upon induction, PRSS55, ADAM2 and EGFP present in supernatants with reduction in pellets. ATP5A1 is shown as a loading control. **b,c** Testicular cells and mature sperm isolated from pAcr-SP-NTP-EGFP transgenic mice, in which EGFP is present within the sperm acrosomes, were digested with various concentrations of proteinase K, and analyzed by immunoblotting for ADAM2, EGFP and PRSS55. ADAM2 and EGFP were examined as positive controls for testicular cell surface and intracellular proteins, respectively. One representative experiment out of three is shown.


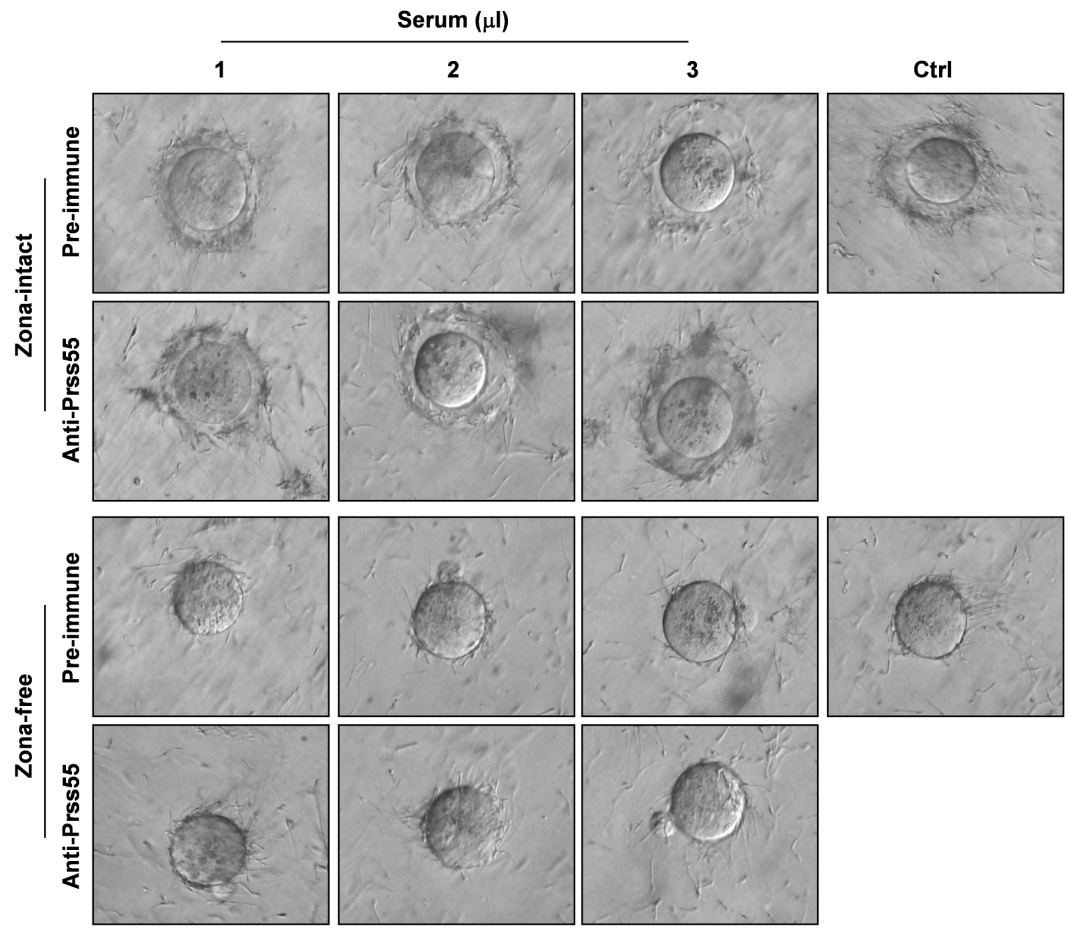


**Fig. S7** Anti-PRSS55 serum is unable to block the binding of wt sperm with either zona-intact or zona-free oocytes. The mouse eggs were incubated with the indicated volume (μl) of antibody or control serum for 30 min, and then capacitated sperm were added and incubated for another 30 min at 37°C. No significant difference was detected between two groups. This experiment was repeated more than three times and the images of a representative experiment are shown.


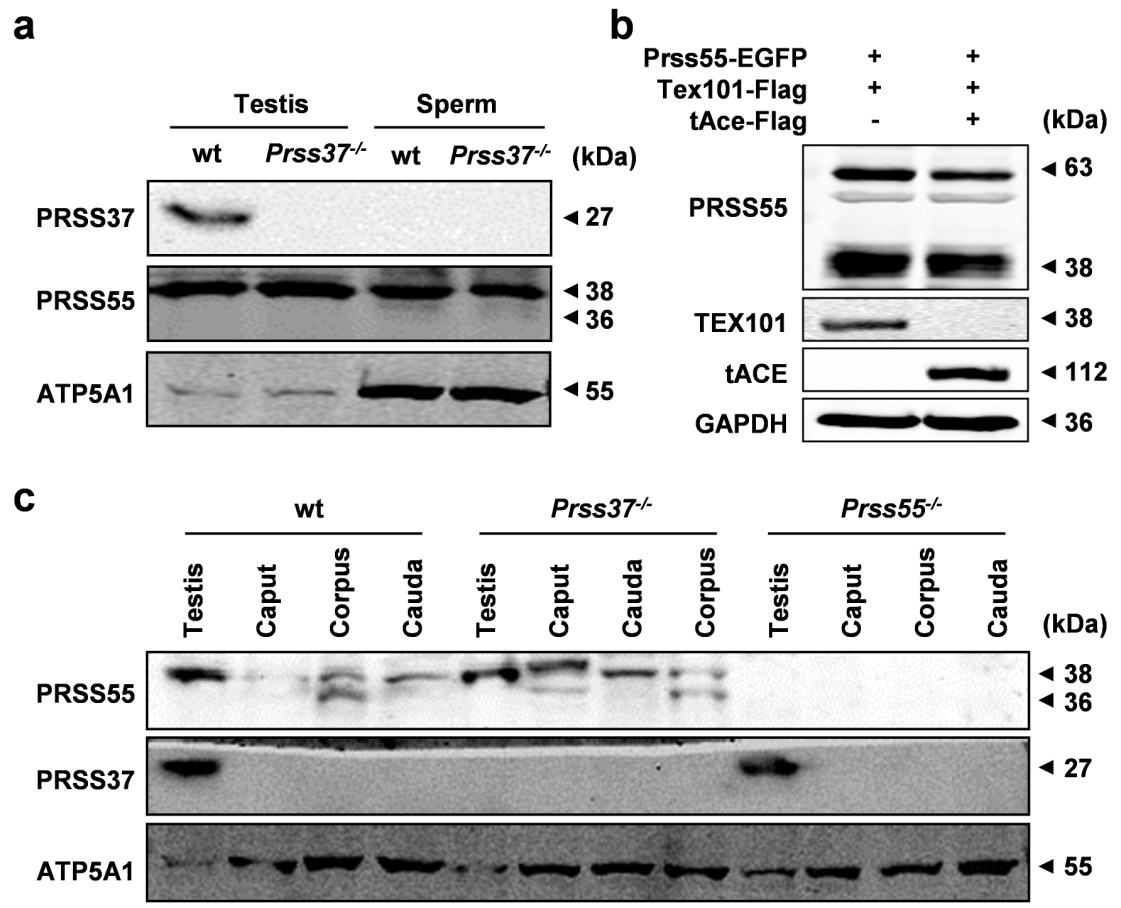


**Fig. S8** PRSS55 is not regulated by tACE or PRSS37. **a** The total lysates of testis and sperm from wt and *Prss37^-/-^* mice were subjected to Western blotting. ATP5A1 serves as a protein loading control. It seems there is no effect of *Prss37* deficiency on PRSS55 protein levels in both testis and sperm. **b** PRSS55, TEX101, and tACE were transiently expressed in HEK293 cells. PRSS55 remains unchanged by the coexpression with tACE, while the TEX101 was abolished when coexpressed with tACE. GAPDH is shown as a loading control. **c** Different protein bands of PRSS55 were detected in caput, corpus and cauda epididymis and the forms of PRSS55 in *Prss37^-/-^* mice are not affected.


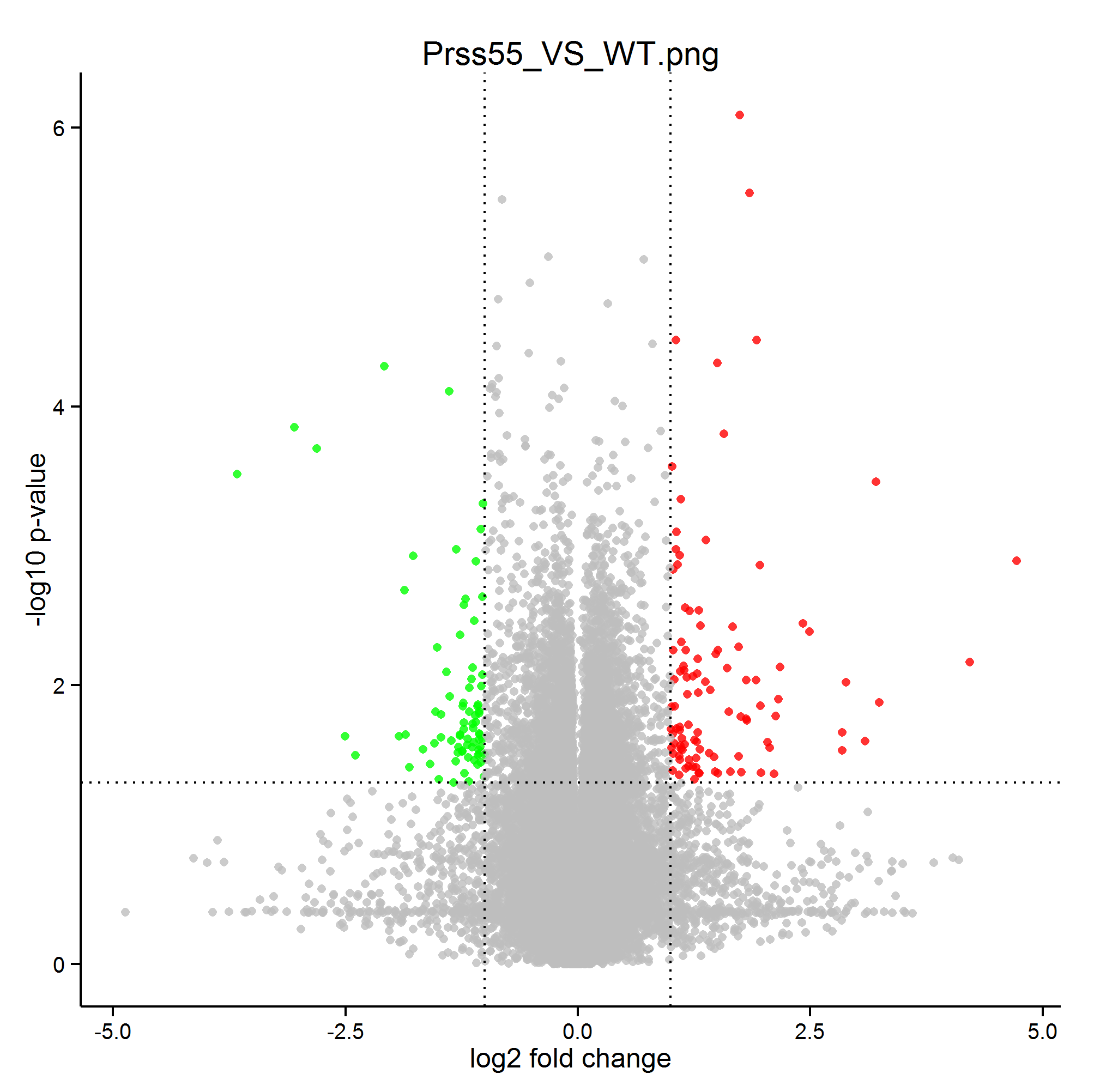


**Fig. S9** The volcano plot of the microarray data. The x-axis shows the log_2_ fold change between *Prss55*-KO testes and wt testes and the y-axis shows the negative log_10_ p-value. Red dots represent differentially expressed (DE) genes (p < 0.05, fc ≥ 2) and green dots represent DE genes (p < 0.05, fc ≤ -2).

**Table S1.** Primers used in PCR assays.

| Primer | Sequence |
| --- | --- |
| P1 | 5’-ATCCTATCAATATGTGGTGAGCATTA-3’ |
| P2 | 5’-CCTCCCCCGTGCCTTCCTTGAC-3’ |
| P3 | 5’-CTGAGCCCAGAAAGCGAAGGA-3’ |
| P4 | 5’-TTCTCTTACAGAGTGCTTTAACCTCA-3’ |
| P5 | 5’-CCATGGGCTCTTTCCTATCA-3’ |
| P6 | 5’-AGCGTTCTGAAGGAGGTGAA-3’ |
| P7 | 5’-TCGCCTTCTTGACGAGTTCT-3’ |
| Prss55-RT-F | 5’-GTGTGAGACCCCTGTATG-3’ |
| Prss55-RT-R | 5’-ACCATATGAGGCACACAGC-3’ |
| Prss55-qRT-R | 5’-GACTCTGAGATCTGTTGGG-3’ |
| β-Actin-F | 5’-TACCCAGGCATTGCTGACAGG-3’ |
| β-Actin-R | 5’-ACTTGCGGTGCACGATGGA-3’ |
| Serpine3-F | 5’-GGACCACAAGGCTGAAGAGAG-3 |
| Serpine3-R | 5’-GACCTGGTACCCTCCTCTGAA-3’ |
| Sult4a1-F | 5’-TGCCAACGTGCTTTTCCTTA-3’ |
| Sult4a1-R | 5’-GGTCACACTTCCCCATCTTCTG-3’ |
| A530072M11Rik-F | 5’-CTTGCCATTGAAGAGGTAAAACT-3’ |
| A530072M11Rik-R | 5’-GGCACTTGGGTATCTTTCTTCC-3’ |
| Hmgxb4-F | 5’-AAGCATCCAGCGCAGAAGGT-3’ |
| Hmgxb4-R | 5’-AAGCTTCCAGACACTGCCACC-3’ |
| Greb1-F | 5’-ACAGGTCTTTCCACATCACATC-3’ |
| Greb1-R | 5’-TGGAGCTCACATAACAAACAGA-3’ |
| Dlgap1-F | 5’-GCAAGCTAATGGAGAGAGAAGA-3’ |
| Dlgap1-R | 5’-CAACTGCAGCATGTCCCAA-3’ |
| Pnoc-F | 5’-GTAATGTGTAGCCAGAAGGAGC-3’ |
| Pnoc-R | 5’-GCAGAAGATGAGTGAGAAGATGG-3’ |
| Dock5-F | 5’-CCTCCTCCAACTCTTCTGACAA-3’ |
| Dock5-R | 5’-GAGACTCTTTGGCCTTTGTGTT-3’ |
| Bloc1s6-F | 5’-CTAGATACACTGGAACAAGAGATTT-3’ |
| Bloc1s6-R | 5’-CGTTGCTGCTCCCTCTCCA-3’ |
| Ltk-F | 5’-TGCCCTACCCTGGACATACC-3’ |
| Ltk-R | 5’-GTGAGTTCAGCACATCAGGGTC-3’ |
| pEGFP-Prss55-F | 5’-ATACTCGAGGCCACCATGATCCTGCCCTCCATCCTGCT-3’ |
| pEGFP-Prss55-R | 5’-ATAAAGCTTGGTTTCCAGTTGGACAAGGCTCTAAG-3’ |

**Table S2.** Comparable motility of capacitated cauda epididymal sperm from wt and *Prss55^-/-^* mice.

| CASA parameters | wt  (n = 5) | *Prss55^-/-^*  (n = 5) |
| --- | --- | --- |
| Total motility (%)  Progressive motility (%)  Rapid motility (%)  Static cell (%)  Path velocity (μm/sec)  Prog. Velocity (μm/sec)  Track speed (μm/sec)  Lateral amplitude (μm)  Beat frequency (Hz)  Straightness (%)  Linearity (%)  Elongation (%) | 94.1 ± 0.7  62.8 ± 2.8  71.8 ± 2.9  5.9 ± 0.7  142.1 ± 3.5  110.2 ± 3.0  222.6 ± 5.0  10.5 ± 0.5  13.9 ± 0.9  76.9 ± 0.6  51.3 ± 0.6  96.3 ± 0.5 | 96.7 ± 0.5  67.7 ± 1.7  78.0 ± 1.4  3.3 ± 0.5  151.2 ± 5.4  115.9 ± 3.4  239.8 ± 9.4  11.3 ± 0.5  14.1 ± 0.4  76.5 ± 0.9  50.5 ± 0.8  97.0 ± 0.3 |

Note: CASA was performed on cauda epididymal sperm from 10-12-week-old wt (n = 5) and *Prss55^-/-^* (n = 5) males as described in materials and methods. Results are expressed as mean ± SE. No significant differences in all parameters were found between two genotypes.

**Table S3.** Differentially expressed (DE) genes (p < 0.05, fc ≥ 2) in the testis of *Prss55^-/-^* mice identified by microarrary analysis.

|  | **Fold change** | **p-value** | **EntrezGene ID** | **Gene name** |
| --- | --- | --- | --- | --- |
| Serpine3 | 26.35 | 1.28E-03 | 319433 | Serpin peptidase inhibitor, clade E (nexin, plasminogen activator inhibitor type 1), member 3 |
| Kcnab1 | 9.25 | 3.47E-04 | 16497 | Potassium voltage-gated channel, shaker-related subfamily, beta member 1 |
| Defa20 | 8.53 | 2.52E-02 | 68009 | Defensin, alpha, 20 |
| Hmgxb4 | 7.18 | 2.19E-02 | 70823 | HMG box domain containing 4 |
| Olfr1184 | 5.63 | 4.12E-03 | 258820 | Olfactory receptor 1184 |
| Sult4a1 | 4.51 | 7.39E-03 | 29859 | Sulfotransferase family 4A, member 1 |
| Fsbp | 4.31 | 4.33E-02 | 100503583 | Fibrinogen silencer binding protein |
| A530072M11Rik | 3.89 | 1.38E-03 | 100415915 | RIKEN cDNA gene A530072M11 |
| H2-Bl | 3.78 | 9.19E-03 | 14963 | Histocompatibility 2, blastocyst |
| Greb1 | 3.59 | 2.93E-06 | 268527 | Gene regulated by estrogen in breast cancer protein |
| LOC102642189 | 3.52 | 1.73E-02 | 102642189 | Uncharacterized LOC102642189 |
| Actl6b | 3.32 | 3.24E-02 | 83766 | Actin-like 6B |
| Vmn2r29 | 3.17 | 3.81E-03 | 76229 | Vomeronasal 2, receptor 29 |
| Trpc7 | 3.13 | 4.16E-02 | 26946 | Transient receptor potential cation channel, subfamily C, member 7 |
| Myo7b | 3.05 | 7.52E-03 | 17922 | Myosin VIIB |
| Abcb11 | 2.97 | 1.57E-04 | 27413 | ATP-binding cassette, sub-family B (MDR/TAP), member 11 |
| Baiap2l2 | 2.84 | 4.29E-02 | 207495 | BAI1-associated protein 2-like 2 |
| Slc4a10 | 2.84 | 5.59E-03 | 94229 | Solute carrier family 4, sodium bicarbonate cotransporter-like, member 10 |
| Dlgap1 | 2.83 | 4.87E-05 | 224997 | Discs, large (Drosophila) homolog-associated protein 1 |
| Phyhip | 2.76 | 3.26E-02 | 105653 | Phytanoyl-CoA hydroxylase interacting protein |
| Zfp521 | 2.60 | 9.12E-04 | 225207 | Zinc finger protein 521 |
| Supt16 | 2.49 | 2.87E-02 | 114741 | Suppressor of Ty 16 |
| Lepr | 2.47 | 4.30E-02 | 16847 | Leptin receptor |
|  | **Fold change** | **p-value** | **EntrezGene ID** | **Gene name** |
| Spire1 | 2.45 | 1.13E-02 | 68166 | Spire homolog 1 (Drosophila) |
| Kcnh5 | 2.45 | 6.47E-03 | 238271 | Potassium voltage-gated channel, subfamily H (eag-related), member 5 |
| Lrrc8e | 2.39 | 4.72E-02 | 72267 | Leucine rich repeat containing 8 family, member E |
| LOC102638889 | 2.39 | 2.48E-02 | 102638889 | Uncharacterized LOC102638889 |
| Mettl3 | 2.36 | 3.86E-02 | 56335 | Methyltransferase like 3 |
| Tmco6 | 2.25 | 8.81E-03 | 71983 | Transmembrane and coiled-coil domains 6 |
| 2300010F08Rik | 2.24 | 5.61E-03 | 71875 | RIKEN cDNA 2300010F08 gene |
| Sptb | 2.24 | 3.96E-02 | 20741 | Spectrin beta, erythrocytic |
| Ly75 | 2.23 | 2.77E-03 | 17076 | Lymphocyte antigen 75 |
| Afm | 2.22 | 2.67E-02 | 280662 | Afamin |
| 5330427O13Rik | 2.17 | 2.42E-02 | 78271 | RIKEN cDNA 5330427O13 gene |
| Fbln1 | 2.17 | 4.87E-03 | 14114 | Fibulin 1 |
| LOC102637912 | 2.16 | 4.65E-04 | 102637912 | Sperm motility kinase W-like |
| Ocstamp | 2.14 | 2.10E-02 | 74614 | Osteoclast stimulatory transmembrane protein |
| Adi1 | 2.14 | 1.17E-03 | 104923 | Acireductone dioxygenase 1 |
| Micall1 | 2.11 | 1.36E-03 | 27008 | Microtubule associated monooxygenase, calponin and LIM domain containing -like 1 |
| Dtwd2 | 2.09 | 7.96E-04 | 68857 | DTW domain containing 2 |
| Ermard | 2.09 | 2.05E-02 | 381062 | ER membrane associated RNA degradation |
| Zfp386 | 2.08 | 3.33E-05 | 56220 | Zinc finger protein 386 (Kruppel-like) |
| Fras1 | 2.07 | 1.41E-02 | 231470 | Fraser syndrome 1 homolog (human) |
| Zfp85os | 2.03 | 2.24E-02 | 68271 | Zinc finger protein 85, opposite strand |
| Pnoc | 2.02 | 2.69E-04 | 18155 | Prepronociceptin |
| Olfr136 | 2.01 | 1.42E-02 | 258803 | Olfactory receptor 136 |
| Cog5 | 2.00 | 2.06E-02 | 238123 | Component of oligomeric golgi complex 5 |
| Ltk | -12.70 | 3.06E-04 | 17005 | Leukocyte tyrosine kinase |
|  | **Fold change** | **p-value** | **EntrezGene ID** | **Gene name** |
| Pla2g4e | -8.28 | 1.41E-04 | 329502 | Phospholipase A2, group IVE |
| Bloc1s6 | -7.01 | 1.99E-04 | 18457 | Biogenesis of lysosomal organelles complex-1, subunit 6, pallidin |
| Gm14123 | -5.25 | 3.18E-02 | 100415913 | Predicted gene 14123 |
| Dock5 | -4.24 | 5.14E-05 | 68813 | Dedicator of cytokinesis 5 |
| Gpr115 | -3.79 | 2.33E-02 | 78249 | G protein-coupled receptor 115 |
| 9430085L16Rik | -3.64 | 2.07E-03 | 77375 | RIKEN cDNA 9430085L16 gene |
| Kcnf1 | -2.92 | 2.62E-02 | 382571 | Potassium voltage-gated channel, subfamily F, member 1 |
| Cyp2a4 | -2.85 | 5.36E-03 | 13086 | Cytochrome P450, family 2, subfamily a, polypeptide 4 |
| LOC102636446 | -2.60 | 7.79E-05 | 102636446 | Uncharacterized LOC102636446 |
| Gpr37l1 | -2.60 | 1.20E-02 | 171469 | G protein-coupled receptor 37-like 1 |
| Olfr1362 | -2.57 | 2.49E-02 | 258739 | Olfactory receptor 1362 |
| Oxa1l | -2.53 | 4.97E-02 | 69089 | Oxidase assembly 1-like |
| Daam1 | -2.37 | 2.98E-02 | 208846 | Dishevelled associated activator of morphogenesis 1 |
| 1700056I18Rik | -2.34 | 1.86E-02 | 73393 | RIKEN cDNA 1700056I18 gene |
| Card10 | -2.33 | 4.26E-02 | 105844 | Caspase recruitment domain family, member 10 |
| Scara5 | -2.17 | 2.56E-02 | 71145 | Scavenger receptor class A, member 5 (putative) |
| Mrgpra2b | -2.16 | 3.46E-02 | 235712 | MAS-related GPR, member A2B |
| Cd300lh | -2.14 | 1.84E-02 | 382551 | CD300 antigen like family member H |
| Serpini2 | -2.09 | 2.90E-02 | 67931 | Serine (or cysteine) peptidase inhibitor, clade I, member 2 |
| Fntb | -2.09 | 3.10E-02 | 110606 | Farnesyltransferase, CAAX box, beta |
| LOC433503 | -2.08 | 2.26E-02 | 433503 | Uncharacterized LOC433503 |
| Pkd2l2 | -2.05 | 3.02E-02 | 53871 | Polycystic kidney disease 2-like 2 |
| Serinc4 | -2.03 | 2.32E-03 | 574418 | Serine incorporator 4 |
| Fosb | -2.03 | 4.98E-04 | 14282 | FBJ osteosarcoma oncogene B |

**Table S4.** Validation of microarray data by qRT-PCR.

| **Gene symbol** | **EntrezGene ID** | **Fold change (microarray)** | **Fold change (qRT-PCR)** |
| --- | --- | --- | --- |
| Serpine3 | 319433 | 26.35 | 198.85 |
| Sult4a1 | 29859 | 4.51 | 13.45 |
| A530072M11Rik | 100415915 | 3.89 | 6.55 |
| Hmgxb4 | 70823 | 7.18 | 3.03 |
| Greb1 | 268527 | 3.59 | 2.09 |
| Dlgap1 | 224997 | 2.83 | 2.27 |
| Pnoc | 18155 | 2.02 | 2.03 |
| Dock5 | 68813 | -4.24 | -2.5 |
| Bloc1s6 | 18457 | -7.01 | -4.82 |
| Ltk | 17005 | -12.70 | -8.01 |
